# U.S. Pediatric Infectious Diseases, 1990–2023 (GBD 2023): Trends in Incidence, Mortality, and DALYs: A Global Burden of Disease Study

**DOI:** 10.1016/j.focus.2026.100535

**Published:** 2026-07-22

**Authors:** Nam T. Nguyen, Quang Dai La, Samrithaa Balakrishnan, Ketan Tamirisa, Jae-Hee Bae, Kavan Shah

**Affiliations:** 1 University of California, San Diego, San Diego, California; 2 Department of Biology, Texas A&M University, College Station, Texas; 3 Rice University, Houston, Texas; 4 Washington University in St. Louis, St. Louis, Michigan; 5 Yale University, New Haven, Connecticut; 6 Cooper Medical School of Rowan University, Camden, New Jersey

**Keywords:** Global Burden of Disease (GBD), infectious disease trends, children 0–14 years, U.S., disability-adjusted life years (DALYs), pediatric epidemiology

## Abstract

**Introduction:**

Infectious disease mortality among children in the U.S. has declined substantially over recent decades, but contemporary national patterns of infectious disease morbidity, mortality, and disability remain incompletely characterized. This study analyzed national trends in infectious disease incidence, mortality, and disability among children aged 0–14 years in the U.S. from 1990 to 2023 using Global Burden of Disease 2023 estimates, focusing on communicable diseases and excluding both COVID-19 and unspecified infectious conditions.

**Methods:**

This retrospective analysis used Global Burden of Disease 2023 age- and sex-specific estimates for deaths, incidence, and disability-adjusted life years, summarized using age-standardized incidence rates; age-standardized mortality rates; and age-standardized disability-adjusted life-year rates per 100,000. In this study, sex refers to male and female categories provided in the GBD 2023 estimates. Temporal trends were quantified using the estimated annual percentage change from log-linear models. Because estimated annual percentage change assumes a constant log-linear trend across the full study period, segmented log-linear regression was conducted for selected high-burden and rare disease categories to assess whether trends varied across distinct time periods.

**Results:**

From 1990 to 2023, infectious disease incidence, mortality, and disability-adjusted life year rates among U.S. children declined across most categories. Enteric infections showed the most rapid incidence reduction (estimated annual percentage change= −10.05), with age-standardized incidence rate decreasing from 17,451.57 to 1,588.48 per 100,000. Neglected tropical diseases and malaria showed a positive relative incidence trend, but the absolute incidence remained extremely low, increasing from 0.07 to 0.14 per 100,000. Regarding mortality and disability-adjusted life years, HIV/AIDS showed the largest declines: deaths decreased from approximately 371 in 1990 to 41 in 2023, and age-standardized disability-adjusted life-year rate decreased from 55.73 to 6.57 per 100,000. Children aged <5 years continued to experience the highest burden, with small but persistent sex-based differences for specific infections. Segmented analyses showed that selected disease categories had nonconstant temporal patterns, supporting the interpretation of estimated annual percentage change as a summary trend measure rather than a complete description of year-to-year change.

**Conclusions:**

U.S. pediatric infectious disease burden declined substantially from 1990 to 2023, driven largely by reductions in major infection categories. However, rare low-burden conditions, such as neglected tropical diseases and malaria, require cautious interpretation because small absolute changes can produce large relative trends. Sustained vaccination coverage, early childhood prevention, and targeted surveillance for travel- or migration-associated infections remain important for maintaining progress and identifying emerging infectious disease signals.

## HIGHLIGHTS

• U.S. pediatric infectious disease burden declined substantially from 1990 to 2023.
• Enteric infections showed the steepest incidence decline among disease groups.
• HIV/AIDS had the largest reductions in pediatric mortality and disability-adjusted life years.
• Children aged <5 years continued to bear the highest infectious disease burden.
• Rare neglected tropical disease and malaria trends require cautious interpretation and surveillance.

## INTRODUCTION

Infectious diseases have remained a substantial source of morbidity among U.S. children, even though pediatric mortality has declined since the 1990s. A comprehensive analysis of recent trends in incidence, mortality, and disability among children aged 0–14 years can help inform prevention priorities and evaluate child health policy priorities. The Global Burden of Disease (GBD) 2023 study provides standardized estimates of disease burden across causes, years, age groups, and sexes, enabling long-term comparisons of infectious disease patterns among U.S. children.^1^

There have been major immunization milestones since the 1990s, which have driven large declines in many vaccine-preventable diseases. For instance, the Vaccines for Children (VFC) program, launched in 1994, has removed cost barriers to important childhood vaccines.^2^ Among the U.S. birth cohorts from 1994 to 2023, routine immunization has prevented approximately 508 million illnesses, 32 million hospitalizations, and more than 1.1 million deaths.^3^ The successive introductions of pneumococcal and rotavirus vaccines in 2000 and 2006 have been associated with reductions in pneumococcal disease and rotavirus-related disease burden among U.S. children, respectively, and provide important context for interpreting broader declines in lower respiratory and enteric infections.^4^, ^5^, ^6^ In addition, immunization has contributed to reductions in otitis media, hepatitis A, and other acute infections.^7^^,^^8^

Despite these advances in disease prevention in the U.S., infectious diseases continue to impose a health burden on children. For instance, congenital syphilis has risen sharply in the past decade, with over 3,700 cases reported in 2022 alone.^9^ Tuberculosis incidence in young children has also recently increased after years of decline.^10^ Meanwhile, neglected tropical diseases and malaria remain rare among U.S. children but remain important for surveillance because some infections are travel associated, are poverty associated, or are affected by changing vector ecology.^11^^,^^12^ These patterns suggest that long-term national progress can coexist with persistent or emerging low-burden infectious disease signals.

Although prior studies have described the infectious disease burden globally or in adult populations, fewer analyses have focused specifically on long-term U.S. pediatric infectious disease trends using the most recent GBD estimates. This study used GBD 2023 data to examine national trends in incidence, mortality, and disability-adjusted life years (DALYs) for major infectious disease categories among U.S. children aged 0–14 years from 1990 to 2023, with additional analyses by age and sex. Segmented regression was also used for selected disease categories to assess whether long-term estimated annual percentage change (EAPC) estimates mask nonconstant temporal patterns.

## METHODS

### Study Population

This was a retrospective study analyzing the changes in incidence, mortality, and DALYs of infectious diseases among children aged 0–14 in the U.S. from 1990 to 2023, using data from the GBD 2023 database. Data were extracted from the Institute for Health Metrics and Evaluation GBD Results Tool (https://vizhub.healthdata.org/gbd-results/) with location set to the U.S.; years set to 1990–2023; age set to 0–14 years (and age subgroups <5, 5–9, and 10–14 years); and sex set to both, male, and female. The GBD database provided age- and sex-specific disease burden estimates. Because the database is publicly available online, the data downloaded do not contain any personal information and do not require ethical IRB approval. Estimates were extracted for the measures of incidence, deaths, and DALYs, and both number and rate outputs were retrieved as reported by GBD. In addition, sex-specific and both-sex estimates were extracted directly from GBD rather than being calculated by averaging male and female estimates. Because the U.S. population aged 0–14 years has an approximately balanced male-to-female distribution, the GBD both-sex rate may visually approximate the midpoint between male and female rates, particularly for rare disease categories with small absolute differences.

The GBD 2023 data set includes 463 health outcomes and risk factors, which are categorized into 3 main groups: communicable, maternal, neonatal, and nutritional disorders; noncommunicable diseases; and injuries. Consistent with prior GBD-based pediatric studies, this study focused exclusively on the communicable disease group, excluding coronavirus disease 2019 (COVID-19) and any other unclassified or unspecified infectious conditions. The infectious disease groups included in this study were HIV/AIDS, sexually transmitted infections excluding HIV, tuberculosis, upper respiratory infections, lower respiratory infections, otitis media (ICD-10: H65–H75.83), enteric infections, neglected tropical diseases and malaria, acute hepatitis, and other infectious diseases. Children are defined as individuals aged 0–14 years, further divided into 3 subgroups: those aged <5 years, those aged 5–9 years, and those aged 10–14 years.^13^

### Measures

The primary outcomes were age-standardized incidence rates (ASIRs), age-standardized mortality rates (ASMRs), and age-standardized DALY rates (ASDRs) per 100,000 population. Absolute numbers of cases, deaths, and DALYs were retained as secondary descriptive measures. Age standardization was performed using the GBD standard population to reduce the influence of demographic structure on temporal comparisons. From the GBD data, DALYs reflect total health loss, integrating both premature mortality and years lived with disability.

### Statistical Analysis

Temporal trends were assessed using the EAPC and 95% CIs, calculated as 100 × (exp[β] − 1), where β represents the annual change in the natural logarithm of the relevant age-standardized rate, including ASIR, ASMR, and ASDR.^13^ EAPC models were fitted using the central GBD estimates, and GBD uncertainty intervals (UIs) were reported descriptively but were not propagated through the log-linear trend models. Because GBD estimates represent model-smoothed annual values rather than independent yearly observations, positive serial autocorrelation in EAPC residuals is expected. Autocorrelation was assessed using the Durbin–Watson test and was detected across most disease categories; among the models with positive serial autocorrelation, Durbin–Watson statistics ranged from 0.05 to 0.77. Accordingly, EAPC estimates and associated CIs are interpreted as descriptive summary trend measures rather than formal hypothesis tests of year-to-year change.

Because EAPC assumes a constant log-linear trend over the full study period, segmented log-linear regression was conducted as a sensitivity analysis for 4 disease categories of a priori clinical and methodologic interest: enteric infections, lower respiratory infections, upper respiratory infections, and neglected tropical diseases and malaria. These categories were selected to represent high-burden respiratory and enteric infections, major contributors to pediatric infectious disease morbidity or mortality, and a rare disease category for which relative trend estimates may be unstable. Models were fitted using the segmented R package (Version 2.1-3) with log-linear base models and allowed up to 3 breakpoints. Models were compared using the Bayesian Information Criterion, which penalizes model complexity; the model with the lowest Bayesian Information Criterion was selected. Specifically, the number of breakpoints retained ranged from 0 to 3 across disease categories and is reported in Table A.7 (available online). Annual percentage change (APC) was estimated for each segment. APC 95% CIs were derived from the SEs of the segment-specific slopes on the log-rate scale and were transformed as 100 × (exp[β] − 1). Selected contextual milestones, including the VFC program in 1994, pneumococcal conjugate vaccine introduction in 2000, rotavirus vaccine introduction in 2006, and COVID-19 pandemic onset in 2020, were displayed descriptively in Figures A.1–A.3 (available online) but were not modeled as causal exposures.^2^^,^^4^

All statistical analyses and data validations were conducted using R (Version 4.3.2) and ggplot2. Descriptive statistics were also performed for all key variables, with results presented as means and 95% UIs.

## RESULTS

From 1990 to 2023, age-standardized incidence, mortality, and DALY rates declined across most infectious disease categories among U.S. children aged 0–14 years (Figure 1). Incidence declined for most disease categories, whereas acute hepatitis remained relatively stable, and neglected tropical diseases and malaria showed a positive relative incidence trend (Figure 2A). Age-standardized incidence for neglected tropical diseases and malaria increased from 0.07 to 0.14 per 100,000 from 1990 to 2023 (EAPC=4.36, 95% CI=2.99, 5.74), although the absolute incidence remained below 1 per 100,000 throughout the study period (Table 1). Although absolute rates remained very low, incidence for neglected tropical diseases and malaria had no identified inflection points in the segmented regression and was best summarized by a single positive 1990–2023 trend (Table A.7, available online, and Figure A.1, available online). Among the disease categories that declined, enteric infections showed the most rapid reduction, with incidence in 2023 approximately 91.1% lower than in 1990 (EAPC= −10.05, 95% CI= −11.85, −8.21) and the ASIR decreasing from 17,451.57 to 1,588.48 per 100,000 (Table 1). Segmented regression identified 3 inflection points in enteric infection incidence at 1995, 2002, and 2015. The final segment showed an increasing APC of +20.71% per year from 2015 to 2023, although the incidence in 2023 remained substantially lower than the 1990 levels (Table A.7, available online, and Figure A.1, available online).

**Figure 1:**
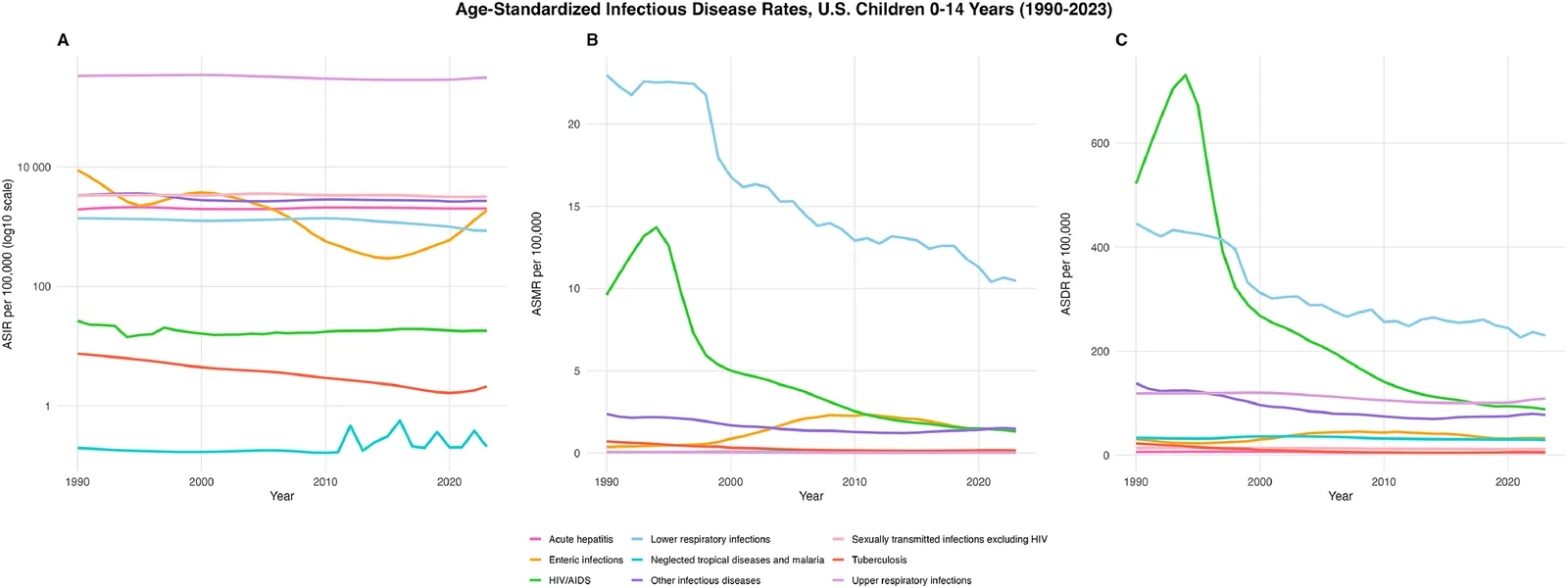
Age-standardized incidence, mortality, and DALY rates of infectious diseases among U.S. children aged 0–14 years, 1990–2023. (A–C) Log 10 scale of (A) ASIR, (B) ASMR, and (C) ASDR. *Note*: Trends in infectious diseases among children in the U.S., 1990–2023. ASDR, age-standardized disability-adjusted life year rate; ASIR, age-standardized incidence rate; ASMR, Age-standardized mortality rate; DALY, disability-adjusted life year.

**Figure 2:**
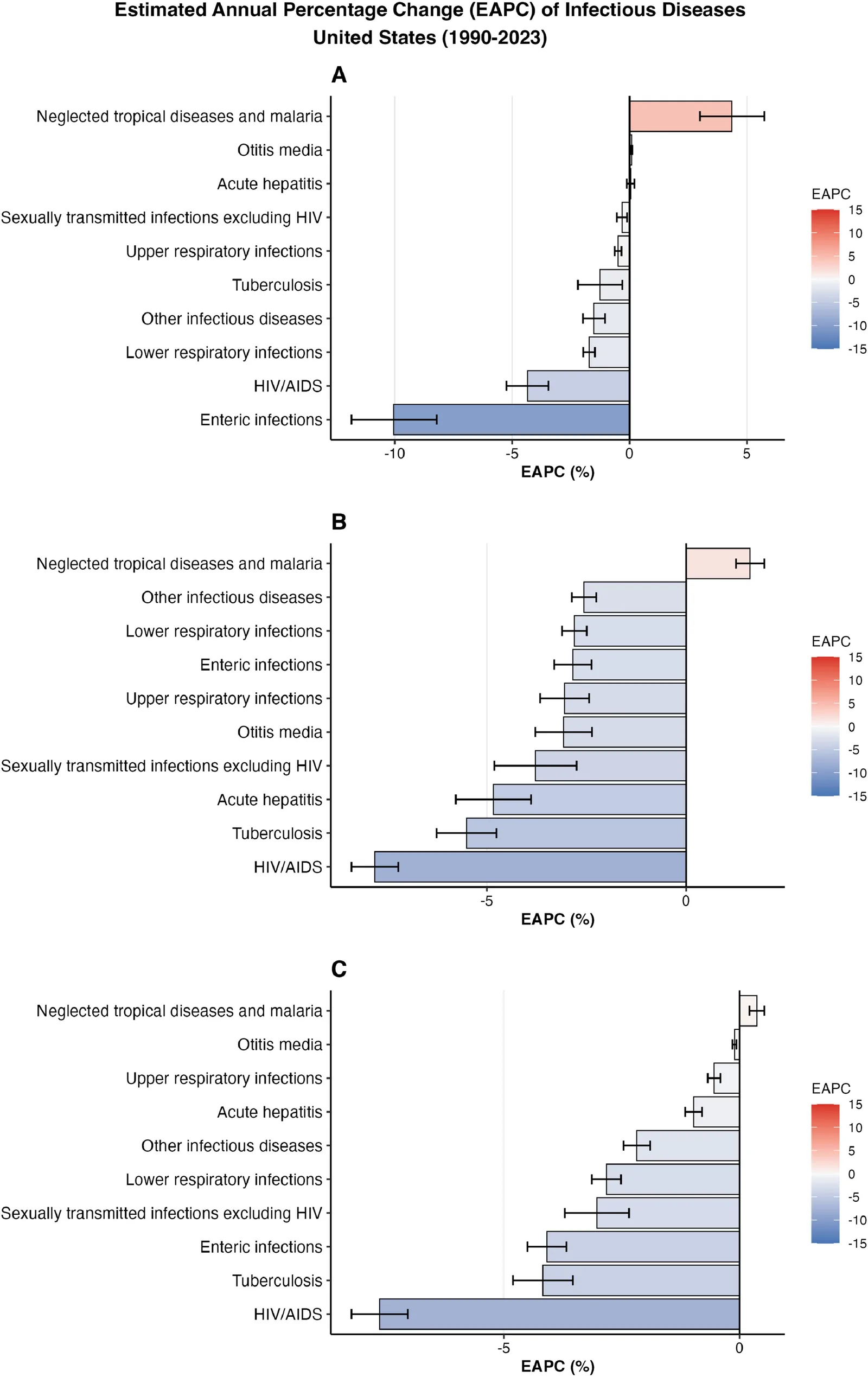
EAPC of infectious diseases in the U.S. from 1990 to 2023 for children aged ≤14 years. (A) ASIR. (B) ASMR. (C) ASDR. *Note*: EAPC in infectious diseases among children in the U.S., 1990–2023, is presented. ASDR, age-standardized disability-adjusted life year rate; ASIR, age-standardized incidence rate; ASMR, Age-standardized mortality rate; EAPC, estimated annual percentage change.

**Table 1:** Age-Standardized Incidence Rates of Infectious Diseases Among Children in the U.S. in 1990 and 2023

Disease	ASIR in 1990 (per 100,000)	ASIR in 2023 (per 100,000)
Acute hepatitis		
Both	3,050.89 (2,500.02; 3,719.85)	3,273.98 (2,677.66; 3,956.29)
Male	3,020.66 (2,470.18; 3,686.32)	3,226.99 (2,632.96; 3,897.54)
Female	3,082.59 (2,512.81; 3,754.64)	3,323.14 (2,715.00; 4,001.98)
Enteric infections		
Both	17,451.57 (11,356.64; 25,725.46)	1,588.48 (1,176.11; 2,082.22)
Male	17,853.04 (11,666.08; 26,208.31)	1,587.96 (1,187.59; 2,084.58)
Female	17,030.51 (11,131.09; 25,043.46)	1,589.03 (1,174.19; 2,067.22)
HIV/AIDS		
Both	18.20 (12.06, 25.47)	4.02 (2.30, 5.82)
Male	18.51 (12.14, 26.02)	4.09 (2.32, 5.96)
Female	17.88 (11.96, 24.91)	3.95 (2.23, 5.68)
Lower respiratory infections		
Both	693.83 (558.49, 857.25)	478.07 (389.95, 581.47)
Male	732.79 (590.09, 903.16)	511.15 (416.56, 618.35)
Female	652.98 (522.43, 808.26)	443.48 (360.65, 542.85)
Neglected tropical diseases and malaria		
Both	0.07 (0.04, 0.12)	0.14 (0.10, 0.20)
Male	0.08 (0.04, 0.16)	0.14 (0.10, 0.20)
Female	0.05 (0.03, 0.09)	0.14 (0.10, 0.19)
Other infectious diseases		
Both	6,681.56 (5,685.40; 7,701.88)	4,454.10 (3,802.87; 5,126.93)
Male	6,628.69 (5,638.58; 7,597.43)	4,360.94 (3,719.93; 5,050.48)
Female	6,737.03 (5,700.40; 7,820.02)	4,551.56 (3,886.46; 5,235.30)
Sexually transmitted infections excluding HIV		
Both	260.01 (197.57, 343.32)	255.04 (198.10, 328.77)
Male	223.71 (159.51, 317.97)	217.55 (161.05, 306.82)
Female	298.17 (222.76, 382.19)	294.44 (228.29, 372.34)
Tuberculosis		
Both	1.47 (0.96, 2.23)	0.66 (0.43, 1.02)
Male	1.13 (0.73, 1.71)	0.71 (0.46, 1.09)
Female	1.82 (1.15, 2.78)	0.62 (0.40, 0.94)
Upper respiratory infections		
Both	456,039.02 (350,801.25; 578,249.27)	453,038.63 (348,422.45; 575,906.47)
Male	452,178.58 (347,872.69; 571,977.20)	451,393.66 (346,819.69; 571,710.11)
Female	460,099.46 (355,391.22; 584,190.95)	454,767.18 (349,257.49; 575,940.96)
Otitis media		
Both	10,889.17 (8,237.13; 1,4152.30)	11,556.74 (8,908.02; 1,4681.98)
Male	11,274.55 (8,565.19; 14,633.59)	11,950.52 (9,201.22; 1,5187.62)
Female	10,485.12 (7,849.04; 13,660.19)	11,145.32 (8,583.25; 14,188.30)

ASIR, age-standardized incidence rate.

EAPC estimates indicated declines in both age-standardized mortality and DALY rates for most disease categories from 1990 to 2023 (Figure 2B and C). HIV/AIDS showed the largest mortality decline, with deaths decreasing from approximately 371 in 1990 to 41 in 2023 and ASMR decreasing by about ninefold (EAPC= −7.81, 95% CI= −8.40, −7.22). Similarly, lower respiratory infections decreased, with approximately 1,538 deaths (UI=1,375; 1,720) in 1990 and 666 deaths (UI=576, 752) in 2023, with about a twofold decrease in ASMR (Table 2). Segmented regression of lower respiratory infection mortality identified a brief period of accelerated decline around 1998–1999 (APC= −15.94%), followed by a sustained moderate decline from 1999 to 2023 (APC= −1.94%) (Table A.7, available online, and Figure A.2, available online). Similarly, HIV/AIDS showed the fastest DALY decline, with ASDR decreasing from 55.73 per 100,000 in 1990 to 6.57 per 100,000 in 2023 (EAPC= −7.64, 95% CI= −8.23, −7.04) (Table 3 and Table A.3, available online).

**Table 2:** Age-Standardized Mortality Rates of Infectious Diseases Among Children in the U.S. in 1990 and 2023

Disease	ASMR in 1990 (per 100,000)	ASMR in 2023 (per 100,000)
Acute hepatitis		
Both	0.02 (0.02, 0.03)	0.01 (0.01, 0.01)
Male	0.02 (0.02, 0.03)	0.01 (0.01, 0.01)
Female	0.02 (0.02, 0.03)	0.01 (0.00, 0.01)
Enteric infections		
Both	0.41 (0.36, 0.47)	0.20 (0.17, 0.23)
Male	0.46 (0.38, 0.56)	0.21 (0.17, 0.26)
Female	0.35 (0.29, 0.44)	0.19 (0.14, 0.24)
HIV/AIDS		
Both	0.64 (0.59, 0.69)	0.07 (0.06, 0.08)
Male	0.72 (0.65, 0.79)	0.07 (0.06, 0.08)
Female	0.56 (0.50, 0.62)	0.07 (0.06, 0.08)
Lower respiratory infections		
Both	2.63 (2.35, 2.94)	1.17 (1.01, 1.32)
Male	2.88 (2.48, 3.42)	1.35 (1.17, 1.58)
Female	2.36 (1.95, 2.76)	0.99 (0.74, 1.21)
Neglected tropical diseases and malaria		
Both	0.04 (0.03, 0.05)	0.05 (0.04, 0.07)
Male	0.04 (0.03, 0.06)	0.05 (0.04, 0.07)
Female	0.03 (0.02, 0.05)	0.05 (0.03, 0.07)
Other infectious diseases		
Both	2.05 (1.87, 2.23)	0.89 (0.79, 0.98)
Male	2.23 (1.97, 2.50)	0.97 (0.83, 1.10)
Female	1.87 (1.66, 2.12)	0.80 (0.68, 0.93)
Sexually transmitted infections excluding HIV		
Both	0.02 (0.02, 0.03)	0.01 (0.00, 0.01)
Male	0.02 (0.02, 0.03)	0.01 (0.00, 0.01)
Female	0.02 (0.02, 0.03)	0.01 (0.00, 0.01)
Tuberculosis		
Both	0.04 (0.03, 0.04)	0.01 (0.01, 0.01)
Male	0.03 (0.03, 0.04)	0.01 (0.00, 0.01)
Female	0.04 (0.03, 0.05)	0.01 (0.01, 0.01)
Upper respiratory infections		
Both	0.10 (0.08, 0.13)	0.03 (0.02, 0.04)
Male	0.11 (0.08, 0.15)	0.04 (0.03, 0.05)
Female	0.09 (0.06, 0.13)	0.02 (0.01, 0.03)
Otitis media		
Both	0.02 (0.01, 0.03)	0.01 (0.00, 0.01)
Male	0.02 (0.01, 0.05)	0.01 (0.00, 0.02)
Female	0.01 (0.00, 0.04)	0.00 (0.00, 0.01)

ASMR, Age-standardized mortality rate.

**Table 3:** ASDRs of Infectious Diseases Among Children in the U.S. in 1990 and 2023

Disease	ASDR in 1990 (per 100,000)	ASDR in 2023 (per 100,000)
Acute hepatitis		
Both	5.43 (3.75, 7.88)	4.40 (2.85, 6.75)
Male	5.39 (3.59, 7.96)	4.43 (2.72, 7.03)
Female	5.46 (3.55, 8.29)	4.37 (2.71, 6.84)
Enteric infections		
Both	69.65 (52.40, 91.92)	20.72 (17.67, 24.37)
Male	74.83 (56.70, 97.97)	21.68 (17.67, 26.07)
Female	64.21 (47.88, 86.18)	19.73 (15.42, 24.77)
HIV/AIDS		
Both	55.73 (51.48, 59.96)	6.57 (5.82, 7.37)
Male	62.42 (56.12, 68.51)	6.46 (5.47, 7.40)
Female	48.71 (43.52, 54.25)	6.70 (5.56, 7.67)
Lower respiratory infections		
Both	232.01 (207.33, 259.46)	102.88 (89.04, 115.99)
Male	254.88 (219.58, 302.12)	118.62 (102.93, 138.38)
Female	208.02 (172.16, 243.25)	86.42 (64.82, 105.93)
Neglected tropical diseases and malaria		
Both	14.29 (6.89, 29.50)	14.37 (9.80, 21.40)
Male	13.52 (6.58, 28.71)	14.60 (7.93, 24.70)
Female	15.10 (6.73, 30.15)	14.13 (7.57, 25.30)
Other infectious diseases		
Both	196.55 (178.15, 218.15)	95.73 (83.83, 109.46)
Male	211.34 (187.54, 238.43)	102.29 (86.21, 118.51)
Female	181.04 (158.72, 209.32)	88.87 (73.69, 105.91)
Sexually transmitted infections excluding HIV		
Both	2.23 (1.75, 2.77)	0.75 (0.54, 1.07)
Male	2.17 (1.60, 2.87)	0.67 (0.45, 0.98)
Female	2.29 (1.60, 3.23)	0.83 (0.52, 1.23)
Tuberculosis		
Both	3.59 (3.14, 4.11)	0.86 (0.70, 1.06)
Male	3.25 (2.72, 3.92)	0.80 (0.64, 1.01)
Female	3.96 (3.28, 4.68)	0.93 (0.73, 1.15)
Upper respiratory infections		
Both	171.66 (102.84, 265.91)	164.55 (95.57, 255.95)
Male	171.42 (102.40, 263.21)	164.80 (95.77, 255.57)
Female	171.92 (102.98, 268.48)	164.28 (95.26, 257.65)
Otitis media		
Both	32.70 (18.82, 54.30)	32.00 (18.29, 53.46)
Male	34.50 (20.00, 57.21)	33.57 (19.26, 57.01)
Female	30.81 (17.68, 50.76)	30.36 (17.09, 50.57)

ASDR, age-standardized disability-adjusted life year rate.

In 2023, lower respiratory infections accounted for the highest number of infectious disease–related deaths among U.S. children, whereas upper respiratory infections had the highest incidence rates (Table 1, Table 2). Neglected tropical diseases and malaria showed positive relative trends in incidence and mortality, whereas DALY rates changed minimally in absolute terms; all of these rates remained low throughout the study period (Figure 3).

**Figure 3:**
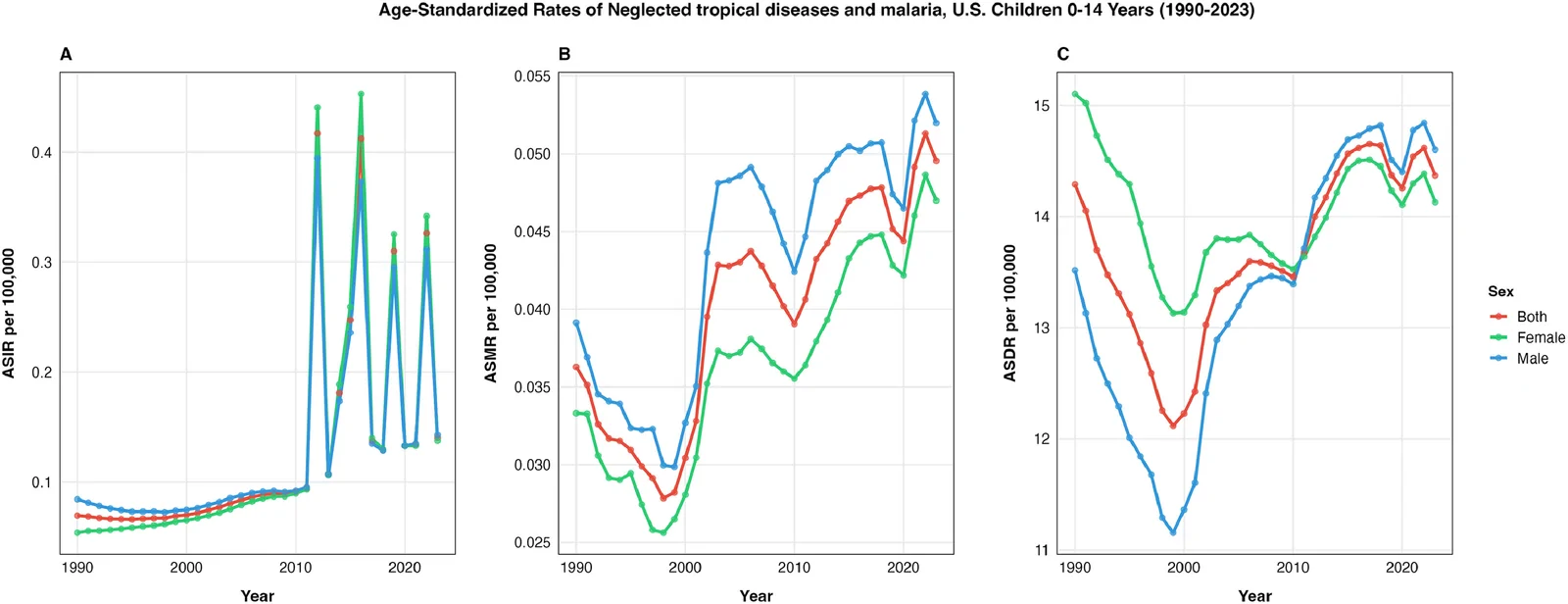
Age-standardized incidence, mortality, and DALY rates of neglected tropical diseases and malaria among U.S. children aged 0–14 years, 1990–2023. (A) ASIR. (B) ASMR. (C) ASDR. *Note*: Presented are trends in the burden of neglected tropical diseases and malaria among children in the U.S., 1990–2023. ASDR, age-standardized disability-adjusted life year rate; ASIR, age-standardized incidence rate; ASMR, Age-standardized mortality rate; DALY, disability-adjusted life year.

Segmented regression revealed that several disease categories exhibited nonlinear temporal patterns that were not fully captured by a single EAPC. Enteric infection incidence followed a multiphase trajectory: it declined sharply in the early 1990s, increased modestly through 2002, declined again through 2015, and reversed upward through 2023, while remaining far below 1990 levels. Lower respiratory infection mortality showed a particularly steep decline in the late 1990s, followed by a sustained moderate decline from 1999 to 2023. Upper respiratory infection trends were comparatively stable in incidence but showed segmented changes in mortality and DALY rates. For neglected tropical diseases and malaria, observed increases occurred against very low absolute rates, supporting an interpretation as a low-burden surveillance signal rather than evidence of a large increase in population-level risk. Full segmented results are presented in Table A.7 (available online) and Figures A.1–A.3 (available online).

In 1990 and 2023, there were no major differences in the burden of infectious disease among children in the U.S. For instance, the ASIR for tuberculosis in 2023 was lower for females than for males (0.62 per 100,000 vs 0.71 per 100,000). In addition, females had a decline in EAPC compared with males’ slight increase (EAPC= −2.76 vs 0.41). For the measured ASMR in 2023, females had lower rates of lower respiratory infections, upper respiratory infections, otitis media, enteric infections, and other infectious diseases, whereas males and females had similar ASMR for other diseases (Table A.2, available online). Overall, females generally had lower ASMR and DALY rate than males, whereas ASIRs were broadly similar between the sexes (Tables A.1–A.3, available online). The study also analyzed the age-specific burden of disease among children across 3 different age groups (i.e., <5 years, 5–9 years, and 10–14 years) from 1990 to 2023. Analyzing the ASIR for infectious diseases in 1990 and 2023, the results demonstrate that acute hepatitis, enteric infections, HIV/AIDS, lower respiratory infections, other infectious diseases, otitis media, tuberculosis, and upper respiratory infections had the highest burden in children aged <5 years. Conversely, the burden of sexually transmitted infections (excluding HIV) and neglected tropical diseases and malaria had the highest burden in the 10–14-year age group (Table A.4, available online).

Regarding the ASMR, the highest mortality rates for all the infectious diseases included were observed in the group of those aged <5 years. In 2023, most infectious disease–related ASMRs were relatively low. However, the ASMR for lower respiratory infections in children aged <5 years was approximately 2.81 per 100,000, notably higher than those of other infectious diseases (Table A.5, available online).

Regarding the ASDR, upper respiratory infections had the highest burden in children aged <5 years in both 1990 and 2023. In general, children who were aged <5 years in the U.S. contributed to a large proportion of the infectious disease burden, and the mortality age groups have declined over the past few decades (Table A.6, available online).

## DISCUSSION

This study presents a comprehensive assessment of infectious disease trends among children aged 0–14 in the U.S. between 1990 and 2023. Overall, incidence, mortality, and DALY rates declined across most infectious disease categories, with the largest reductions in mortality and DALY observed for HIV/AIDS and the steepest reduction in incidence observed for enteric infections. Children aged <5 years continued to experience the highest burden of most infections, whereas sex-specific differences were modest. Segmented analyses showed that selected disease categories had nonconstant temporal patterns, reinforcing that EAPC should be interpreted as a summary measure rather than a complete description of temporal change.

Although there were no major disparities in total disease burden by sex, some infections showed a slightly higher incidence or mortality among male children. For instance, boys had a higher ASIR for tuberculosis and a higher ASMR for multiple conditions, including respiratory and enteric infections. These differences may arise from sex-based immunology. In prior studies, it has been suggested that estrogen can provide protective effects in immune responses, whereas androgens can enhance susceptibility to certain pathogens.^14^^,^^15^ Because these differences were relatively small, sex may not be a primary driver of pediatric infectious disease trends.

The sharp declines in vaccine-preventable diseases in the U.S. highlight the importance of sustained immunization and pediatric prevention infrastructure. For instance, the VFC program, which was launched in 1994, helped eliminate financial barriers to essential vaccinations in millions of children, and it is credited with preventing over 508 million illnesses and around 1.1 million deaths in the birth cohorts from 1994 to 2023.^3^ With coverage levels for routine childhood vaccines (i.e., measles, mumps, and rubella; diphtheria, tetanus, and pertussis; and *Hemophilus influenzae* Type b) consistently near or above 90%, sustaining high coverage remains central to preventing the re-emergence of vaccine-preventable infections.^16^^,^^17^ These findings support continued investment in vaccination delivery, reminder–recall systems, school-entry vaccination monitoring, and primary care access.

In addition, Medicaid and the Children's Health Insurance Program have played an important role in expanding access to preventive care for children across the country, including vaccinations, routine screenings, and treatment.^18^ However, structural inequities persist, and infectious disease outcomes vary across racial, ethnic, socioeconomic, insurance, and geographic lines, where undervaccination can occur among uninsured or underinsured children, especially those in rural areas or from immigrant households.^19^ Recent Centers for Disease Control and Prevention data show that vaccination coverage remains lower among VFC-eligible children than among noneligible children and that among VFC-eligible children born in 2020, uninsured children had coverage that was 18.9–34.7 percentage points lower than that of Medicaid-insured children for selected vaccine measures. These gaps reinforce the need for targeted outreach; reminder–recall systems; school- and community-based vaccination programs; and improved access for uninsured, rural, and immigrant children.^20^^,^^21^

The results indicate that children aged <5 years often bear a disproportionate share of the infectious disease burden. For instance, immature immunity, higher exposure to group settings, and limited verbal communication for early symptom reporting can contribute to a higher risk for children in this age group.^22^^,^^23^ A continued emphasis on early-life intervention strategies (e.g., perinatal screening for infections, routine immunization, and parental education) remains important. The positive relative trends observed for neglected tropical diseases and malaria should be interpreted cautiously. Although age-standardized incidence increased from 0.07 to 0.14 per 100,000, the absolute burden remained extremely low, and segmented analyses showed no identified inflection points for incidence. For rare pediatric outcomes, relative metrics such as EAPC can be unstable and may be influenced by sparse data, reporting changes, coding practices, and GBD modeling assumptions.^24^ These findings should therefore be viewed as a low-burden surveillance signal rather than definitive evidence of a large increase in population-level risk. Clinically, the findings support maintaining travel-history assessment and awareness of imported malaria, given that most U.S. malaria cases are travel associated.^12^ Continued attention to neglected infections and targeted surveillance for children with relevant travel, migration, maternal, or exposure histories remain important, particularly because neglected infections in the U.S. can be concentrated in marginalized or poverty-affected populations.^11^^,^^25^ Pretravel counseling and school- or clinic-based education may also support the prevention of mosquito-borne infections among children.^26^^,^^27^

Taken together, these findings support several practical intervention priorities. First, maintaining high routine childhood vaccination coverage remains essential to preserve long-term declines in respiratory, enteric, hepatitis-related, and otitis-associated disease burdens. Reminder and recall interventions can improve immunization uptake, and school-based vaccination programs can support vaccine delivery in high-income settings.^28^^,^^29^ Second, the persistent concentration of burden among children aged <5 years supports continued investment in early-life prevention, timely pediatric care, and caregiver education. Third, rare but increasing or variable conditions, such as neglected tropical diseases and malaria, support clinician education, travel-related screening, and targeted surveillance rather than broad population-level alarm; the pediatric travel medicine literature emphasizes pretravel counseling, immunization review, and malaria prevention for children traveling internationally.^30^ Finally, reducing insurance- and access-related vaccination gaps should remain central to pediatric infectious disease prevention, because vaccine financing gaps have limited access to recommended vaccines for underinsured children.^31^

### Limitations

This study has several limitations. First, the GBD data are model based and subject to uncertainty, particularly for low-incidence conditions, where the surveillance data may be sparse. Moreover, misclassification, underreporting, and data interpolation may affect the year-to-year trends.^24^ This limitation is especially important for rare outcomes, such as neglected tropical diseases, malaria, tuberculosis, and pediatric deaths from low-frequency infections, where small absolute changes can generate large relative changes. Second, the GBD data set does not provide individual-level information on race, insurance, vaccination, or SES, which limits causal inferences. Consequently, the sex- and age-related comparisons do not account for overlapping factors, such as poverty or housing insecurity, which could have affected infection risks. In addition, this study did not analyze the Social Development Index score at the state level, and the analysis was not stratified by region or state, which may mask local disparities in disease burden. Segmented regression can also identify changes in slope but cannot determine the causes of those changes. Vaccine introductions and the COVID-19 pandemic were displayed only as contextual temporal markers and were not modeled as causal exposures. Finally, Durbin–Watson testing identified strong positive serial autocorrelation in EAPC model residuals across most disease categories, consistent with the modeled annual structure of GBD estimates. CIs should therefore be interpreted as descriptive measures of trend uncertainty rather than as inferential bounds derived from independent yearly observations.

## CONCLUSIONS

In conclusion, the infectious disease burden among children in the U.S. has declined substantially from 1990 to 2023, likely reflecting long-term improvements in immunization programs, pediatric prevention, and healthcare access infrastructure. However, children aged <5 years continue to experience the highest burden, and small but persistent sex-based differences remain for specific infections. Rare, low-burden conditions such as neglected tropical diseases and malaria require cautious interpretation but support continued surveillance, particularly for travel- or migration-associated infections. Sustaining high vaccination coverage, closing access gaps, strengthening early-childhood prevention, and maintaining clinician awareness of low-frequency emerging infections will be essential to preserve progress in reducing the pediatric infectious disease burden.

## CRediT authorship contribution statement

**Nam T. Nguyen:** Conceptualization, Methodology, Data curation, Formal analysis, Visualization, Writing – original draft, Writing – review & editing, Project administration. **Quang Dai La:** Data curation, Formal analysis, Validation, Visualization, Writing – original draft, Writing – review & editing. **Samrithaa Balakrishnan:** Data curation, Formal analysis, Validation, Visualization, Writing – review & editing. **Ketan Tamirisa:** Conceptualization, Data curation, Formal analysis, Visualization, Writing – original draft, Writing – review & editing. **Jae-Hee Bae:** Data curation, Formal analysis, Validation, Writing – original draft, Writing – review & editing. **Kavan Shah:** Conceptualization, Visualization, Writing – original draft, Writing – review & editing, Supervision.

## Appendix Supplementary materials

Image, application 1

## Footnotes

Supplementary material associated with this article can be found in the online version at doi:10.1016/j.focus.2026.100535.
