# Supplementary material for: U.S. Pediatric Infectious Diseases, 1990–2023 (GBD 2023): Trends in Incidence, Mortality, and DALYs: A Global Burden of Disease Study
Source: AJPM Focus. 2026 Jul 22;5(6):100535. doi: 10.1016/j.focus.2026.100535 (PMC13753570; doi:10.1016/j.focus.2026.100535)
Supplement: Supplementary file 1 [file mmc1.pdf]

Appendix Materials

Article title: U.S. Pediatric Infectious Diseases, 1990–2023 (GBD 2023): Trends in Incidence, Mortality, and DALYs: A Global Burden of Disease Study.

Authors: Nam T. Nguyen, BS; Quang Dai La; Samrithaa Balakrishnan, BA; Ketan Tamirisa; Jae-Hee Bae, BS; Kavan Shah, BS

Journal: AJPM FOCUS

Table A.1: Incidence rate burden of infectious diseases among children in the United States

Disease	Sex	Cases in 1990	Cases in 2023	ASR in 1990 (per 100,000)	ASR in 2023 (per 100,000)	EAPC (95% CI)
Acute hepatitis	Both	1722847.47 (1411380.20, 2103071.42)	1934598.51 (1584167.41, 2333736.43)	3050.89 (2500.02, 3719.85)	3273.98 (2677.66, 3956.29)	0.04 (-0.12, 0.21)
Acute hepatitis	Male	873181.10 (713888.17, 1066505.20)	975731.33 (796849.18, 1176557.32)	3020.66 (2470.18, 3686.32)	3226.99 (2632.96, 3897.54)	0.06 (-0.09, 0.22)
Acute hepatitis	Female	849666.37 (692262.05, 1036162.39)	958867.19 (784342.32, 1152856.67)	3082.59 (2512.81, 3754.64)	3323.14 (2715.00, 4001.98)	0.02 (-0.14, 0.19)
HIV/AIDS	Both	10783.21 (7143.05, 15088.57)	2236.28 (1278.82, 3235.28)	18.20 (12.06, 25.47)	4.02 (2.30, 5.82)	-4.35 (-5.24, -3.45)
HIV/AIDS	Male	5612.67 (3683.09, 7892.16)	1162.70 (659.14, 1693.28)	18.51 (12.14, 26.02)	4.09 (2.32, 5.96)	-4.35 (-5.24, -3.45)
HIV/AIDS	Female	5170.54 (3460.18, 7205.73)	1073.57 (605.41, 1544.80)	17.88 (11.96, 24.91)	3.95 (2.23, 5.68)	-4.35 (-5.24, -3.46)
Sexually transmitted infections excluding HIV	Both	137675.25 (104573.82, 181841.22)	160601.40 (124785.14, 206970.17)	260.01 (197.57, 343.32)	255.04 (198.10, 328.77)	-0.32 (-0.54, -0.10)
Sexually transmitted infections excluding HIV	Male	60732.09 (43289.78, 86329.90)	70161.53 (51957.00, 98942.09)	223.71 (159.51, 317.97)	217.55 (161.05, 306.82)	-0.39 (-0.58, -0.19)
Sexually transmitted infections excluding HIV	Female	76943.17 (57465.36, 98657.35)	90439.87 (70141.14, 114338.62)	298.17 (222.76, 382.19)	294.44 (228.29, 372.34)	-0.28 (-0.52, -0.04)
Tuberculosis	Both	826.05 (543.05, 1247.80)	392.51 (252.21, 606.25)	1.47 (0.96, 2.23)	0.66 (0.43, 1.02)	-1.26 (-2.20, -0.31)
Tuberculosis	Male	329.03 (213.44, 494.96)	212.35 (136.87, 329.60)	1.13 (0.73, 1.71)	0.71 (0.46, 1.09)	0.41 (-0.91, 1.75)
Tuberculosis	Female	497.02 (315.88, 755.15)	180.16 (114.76, 275.52)	1.82 (1.15, 2.78)	0.62 (0.40, 0.94)	-2.76 (-3.38, -2.14)

Disease	Sex	Cases in 1990	Cases in 2023	ASR in 1990 (per 100,000)	ASR in 2023 (per 100,000)	EAPC (95% CI)
Lower respiratory infections	Both	400779.19 (323784.94, 493592.47)	275454.67 (223908.33, 335941.67)	693.83 (558.49, 857.25)	478.07 (389.95, 581.47)	-1.72 (-1.97, -1.48)
Lower respiratory infections	Male	216992.37 (175354.70, 266589.89)	150386.68 (122115.43, 182419.53)	732.79 (590.09, 903.16)	511.15 (416.56, 618.35)	-1.72 (-1.98, -1.47)
Lower respiratory infections	Female	183786.82 (147624.42, 226762.32)	125067.98 (101361.91, 153498.61)	652.98 (522.43, 808.26)	443.48 (360.65, 542.85)	-1.72 (-1.96, -1.48)
Upper respiratory infections	Both	258206821.75 (199330427.35, 326436051.84)	266887613.87 (204354536.82, 340367172.69)	456039.02 (350801.25, 578249.27)	453038.63 (348422.45, 575906.47)	-0.49 (-0.64, -0.35)
Upper respiratory infections	Male	131203899.63 (101314429.25, 165464795.27)	135976818.88 (103973147.26, 172775803.58)	452178.58 (347872.69, 571977.20)	451393.66 (346819.69, 571710.11)	-0.49 (-0.63, -0.35)
Upper respiratory infections	Female	127002922.12 (98456648.84, 160800661.48)	130910795.00 (100114162.23, 166337584.66)	460099.46 (355391.22, 584190.95)	454767.18 (349257.49, 575940.96)	-0.49 (-0.64, -0.34)
Otitis media	Both	6295352.71 (4768159.15, 8172811.73)	6641974.82 (5111557.24, 8447516.89)	10889.17 (8237.13, 14152.30)	11556.74 (8908.02, 14681.98)	0.08 (0.05, 0.12)
Otitis media	Male	3340681.84 (2541330.24, 4331921.74)	3506833.81 (2695402.64, 4462236.39)	11274.55 (8565.19, 14633.59)	11950.52 (9201.22, 15187.62)	0.08 (0.05, 0.11)
Otitis media	Female	2954670.87 (2214687.62, 3844190.38)	3135141.01 (2411051.57, 3997173.46)	10485.12 (7849.04, 13660.19)	11145.32 (8583.25, 14188.30)	0.09 (0.05, 0.12)
Enteric infections	Both	10084099.12 (6600535.51, 14798270.83)	900834.33 (663583.78, 1186111.93)	17451.57 (11356.64, 25725.46)	1588.48 (1176.11, 2082.22)	-10.05 (-11.85, -8.21)
Enteric infections	Male	5283720.31 (3473254.12, 7719259.24)	460446.31 (342516.63, 607236.16)	17853.04 (11666.08, 26208.31)	1587.96 (1187.59, 2084.58)	-10.05 (-11.85, -8.21)
Enteric infections	Female	4800378.80 (3156179.93, 7023829.41)	440388.03 (323804.97, 575577.76)	17030.51 (11131.09, 25043.46)	1589.03 (1174.19, 2067.22)	-10.04 (-11.84, -8.21)
Neglected tropical diseases and malaria	Both	37.97 (20.80, 67.50)	85.10 (59.71, 119.34)	0.07 (0.04, 0.12)	0.14 (0.10, 0.20)	4.36 (2.99, 5.74)

Disease	Sex	Cases in 1990	Cases in 2023	ASR in 1990 (per 100,000)	ASR in 2023 (per 100,000)	EAPC (95% CI)
Neglected tropical diseases and malaria	Male	23.55 (12.54, 43.19)	44.40 (30.78, 62.58)	0.08 (0.04, 0.16)	0.14 (0.10, 0.20)	3.73 (2.44, 5.04)
Neglected tropical diseases and malaria	Female	14.42 (8.18, 24.60)	40.70 (28.61, 56.67)	0.05 (0.03, 0.09)	0.14 (0.10, 0.19)	5.10 (3.67, 6.56)
Other infectious diseases	Both	3859812.46 (3288551.48, 4441103.42)	2601259.29 (2220002.55, 2994642.19)	6681.56 (5685.40, 7701.88)	4454.10 (3802.87, 5126.93)	-1.52 (-1.98, -1.05)
Other infectious diseases	Male	1960683.71 (1669963.88, 2243113.35)	1302935.82 (1110845.97, 1509149.87)	6628.69 (5638.58, 7597.43)	4360.94 (3719.93, 5050.48)	-1.53 (-2.00, -1.06)
Other infectious diseases	Female	1899128.75 (1608862.49, 2200391.24)	1298323.48 (1108107.72, 1493561.79)	6737.03 (5700.40, 7820.02)	4551.56 (3886.46, 5235.30)	-1.51 (-1.97, -1.04)

Table A.2: Burden of mortality from infectious diseases among children in the United States

Disease	Sex	Deaths in 1990	Deaths in 2023	ASMR in 1990 (per 100,000)	ASMR in 2023 (per 100,000)	EAPC (95% CI)
Acute hepatitis	Both	13.46 (10.32, 17.18)	4.06 (3.02, 5.47)	0.02 (0.02, 0.03)	0.01 (0.01, 0.01)	-4.84 (-5.78, -3.89)
Acute hepatitis	Male	6.92 (4.83, 10.01)	2.35 (1.62, 3.65)	0.02 (0.02, 0.03)	0.01 (0.01, 0.01)	-4.44 (-5.37, -3.49)
Acute hepatitis	Female	6.54 (4.39, 9.02)	1.71 (1.10, 2.43)	0.02 (0.02, 0.03)	0.01 (0.00, 0.01)	-5.33 (-6.28, -4.36)
HIV/AIDS	Both	371.00 (342.13, 399.17)	41.11 (36.30, 46.10)	0.64 (0.59, 0.69)	0.07 (0.06, 0.08)	-7.81 (-8.40, -7.22)
HIV/AIDS	Male	213.01 (191.37, 233.95)	20.62 (17.29, 23.84)	0.72 (0.65, 0.79)	0.07 (0.06, 0.08)	-8.14 (-8.85, -7.43)
HIV/AIDS	Female	157.99 (141.04, 175.95)	20.50 (16.83, 23.58)	0.56 (0.50, 0.62)	0.07 (0.06, 0.08)	-7.43 (-7.92, -6.95)
Sexually transmitted infections excluding HIV	Both	13.02 (10.48, 15.34)	3.15 (2.52, 3.86)	0.02 (0.02, 0.03)	0.01 (0.00, 0.01)	-3.79 (-4.81, -2.75)
Sexually transmitted infections excluding HIV	Male	6.77 (5.06, 8.76)	1.63 (1.17, 2.14)	0.02 (0.02, 0.03)	0.01 (0.00, 0.01)	-3.89 (-4.98, -2.78)
Sexually transmitted infections excluding HIV	Female	6.25 (4.49, 8.45)	1.51 (1.00, 2.07)	0.02 (0.02, 0.03)	0.01 (0.00, 0.01)	-3.69 (-4.74, -2.62)
Tuberculosis	Both	21.99 (19.21, 25.06)	3.81 (3.23, 4.38)	0.04 (0.03, 0.04)	0.01 (0.01, 0.01)	-5.51 (-6.26, -4.76)
Tuberculosis	Male	10.28 (8.56, 12.43)	1.73 (1.41, 2.08)	0.03 (0.03, 0.04)	0.01 (0.00, 0.01)	-5.30 (-6.04, -4.54)
Tuberculosis	Female	11.70 (9.71, 13.93)	2.08 (1.62, 2.59)	0.04 (0.03, 0.05)	0.01 (0.01, 0.01)	-5.70 (-6.46, -4.93)

Disease	Sex	Deaths in 1990	Deaths in 2023	ASMR in 1990 (per 100,000)	ASMR in 2023 (per 100,000)	EAPC (95% CI)
Lower respiratory infections	Both	1537.92 (1374.70, 1720.46)	666.02 (576.12, 751.76)	2.63 (2.35, 2.94)	1.17 (1.01, 1.32)	-2.81 (-3.12, -2.49)
Lower respiratory infections	Male	864.64 (744.15, 1025.31)	391.49 (338.85, 457.62)	2.88 (2.48, 3.42)	1.35 (1.17, 1.58)	-2.69 (-3.03, -2.34)
Lower respiratory infections	Female	673.28 (557.15, 787.63)	274.52 (206.57, 336.97)	2.36 (1.95, 2.76)	0.99 (0.74, 1.21)	-2.96 (-3.24, -2.68)
Upper respiratory infections	Both	59.50 (46.50, 74.49)	17.26 (13.14, 22.62)	0.10 (0.08, 0.13)	0.03 (0.02, 0.04)	-3.05 (-3.66, -2.44)
Upper respiratory infections	Male	33.55 (23.04, 45.86)	11.21 (7.87, 15.76)	0.11 (0.08, 0.15)	0.04 (0.03, 0.05)	-2.77 (-3.40, -2.13)
Upper respiratory infections	Female	25.95 (18.21, 35.99)	6.05 (4.06, 8.83)	0.09 (0.06, 0.13)	0.02 (0.01, 0.03)	-3.49 (-4.08, -2.89)
Otitis media	Both	10.53 (3.57, 19.59)	3.40 (1.33, 8.36)	0.02 (0.01, 0.03)	0.01 (0.00, 0.01)	-3.08 (-3.79, -2.36)
Otitis media	Male	6.86 (1.90, 14.33)	2.39 (0.83, 7.16)	0.02 (0.01, 0.05)	0.01 (0.00, 0.02)	-2.83 (-3.69, -1.95)
Otitis media	Female	3.67 (0.78, 11.19)	1.01 (0.21, 3.41)	0.01 (0.00, 0.04)	0.00 (0.00, 0.01)	-3.56 (-4.01, -3.12)
Enteric infections	Both	240.56 (210.48, 276.23)	110.86 (93.29, 130.81)	0.41 (0.36, 0.47)	0.20 (0.17, 0.23)	-2.84 (-3.31, -2.37)
Enteric infections	Male	138.28 (115.91, 168.11)	59.77 (47.61, 74.06)	0.46 (0.38, 0.56)	0.21 (0.17, 0.26)	-3.15 (-3.66, -2.64)
Enteric infections	Female	102.28 (84.29, 127.21)	51.09 (38.20, 65.27)	0.35 (0.29, 0.44)	0.19 (0.14, 0.24)	-2.42 (-2.88, -1.96)
Neglected tropical diseases and malaria	Both	21.01 (16.62, 26.93)	28.38 (21.69, 37.57)	0.04 (0.03, 0.05)	0.05 (0.04, 0.07)	1.60 (1.25, 1.96)
Neglected tropical diseases and malaria	Male	11.59 (8.38, 16.33)	15.23 (10.44, 21.04)	0.04 (0.03, 0.06)	0.05 (0.04, 0.07)	1.57 (1.17, 1.97)

Disease	Sex	Deaths in 1990	Deaths in 2023	ASMR in 1990 (per 100,000)	ASMR in 2023 (per 100,000)	EAPC (95% CI)
Neglected tropical diseases and malaria	Female	9.42 (6.70, 12.81)	13.15 (9.47, 18.44)	0.03 (0.02, 0.05)	0.05 (0.03, 0.07)	1.65 (1.32, 1.98)
Other infectious diseases	Both	1197.56 (1092.75, 1300.49)	503.89 (451.23, 556.82)	2.05 (1.87, 2.23)	0.89 (0.79, 0.98)	-2.57 (-2.87, -2.26)
Other infectious diseases	Male	665.85 (590.71, 747.70)	280.30 (239.85, 319.72)	2.23 (1.97, 2.50)	0.97 (0.83, 1.10)	-2.53 (-2.84, -2.22)
Other infectious diseases	Female	531.71 (472.79, 602.86)	223.59 (188.89, 258.21)	1.87 (1.66, 2.12)	0.80 (0.68, 0.93)	-2.61 (-2.92, -2.30)

Table A.3: Burden of DALYs caused by infectious diseases among children in the United States

Disease	Sex	DALYs in 1990	DALYs in 2023	ASDR in 1990 (per 100,000)	ASDR in 2023 (per 100,000)	EAPC (95% CI)
Acute hepatitis	Both	3039.34 (2107.80, 4398.06)	2673.73 (1726.66, 4118.17)	5.43 (3.75, 7.88)	4.40 (2.85, 6.75)	-0.98 (-1.15, -0.80)
Acute hepatitis	Male	1547.79 (1034.19, 2281.77)	1374.62 (841.13, 2185.86)	5.39 (3.59, 7.96)	4.43 (2.72, 7.03)	-0.94 (-1.11, -0.76)
Acute hepatitis	Female	1491.55 (970.87, 2254.55)	1299.11 (803.71, 2039.27)	5.46 (3.55, 8.29)	4.37 (2.71, 6.84)	-1.02 (-1.21, -0.83)
HIV/AIDS	Both	32337.19 (29886.58, 34776.15)	3796.39 (3358.58, 4261.33)	55.73 (51.48, 59.96)	6.57 (5.82, 7.37)	-7.64 (-8.23, -7.04)
HIV/AIDS	Male	18516.42 (16664.20, 20304.90)	1906.14 (1611.65, 2186.91)	62.42 (56.12, 68.51)	6.46 (5.47, 7.40)	-7.96 (-8.67, -7.24)
HIV/AIDS	Female	13820.77 (12360.29, 15385.51)	1890.25 (1567.96, 2166.22)	48.71 (43.52, 54.25)	6.70 (5.56, 7.67)	-7.27 (-7.76, -6.78)
Sexually transmitted infections excluding HIV	Both	1296.22 (1018.36, 1606.03)	438.45 (312.29, 631.79)	2.23 (1.75, 2.77)	0.75 (0.54, 1.07)	-3.03 (-3.71, -2.35)
Sexually transmitted infections excluding HIV	Male	648.25 (480.21, 857.10)	197.58 (130.63, 293.30)	2.17 (1.60, 2.87)	0.67 (0.45, 0.98)	-3.24 (-4.10, -2.36)
Sexually transmitted infections excluding HIV	Female	647.97 (453.25, 909.93)	240.87 (149.61, 357.75)	2.29 (1.60, 3.23)	0.83 (0.52, 1.23)	-2.85 (-3.42, -2.27)
Tuberculosis	Both	2078.82 (1818.36, 2376.91)	496.62 (401.86, 611.10)	3.59 (3.14, 4.11)	0.86 (0.70, 1.06)	-4.17 (-4.81, -3.54)
Tuberculosis	Male	963.60 (808.25, 1163.88)	233.96 (188.86, 296.39)	3.25 (2.72, 3.92)	0.80 (0.64, 1.01)	-3.76 (-4.37, -3.14)
Tuberculosis	Female	1115.22 (925.73, 1317.23)	262.67 (205.35, 327.73)	3.96 (3.28, 4.68)	0.93 (0.73, 1.15)	-4.54 (-5.22, -3.86)

Disease	Sex	DALYs in 1990	DALYs in 2023	ASDR in 1990 (per 100,000)	ASDR in 2023 (per 100,000)	EAPC (95% CI)
Lower respiratory infections	Both	135997.31 (121548.21, 152091.56)	58360.94 (50519.48, 65822.77)	232.01 (207.33, 259.46)	102.88 (89.04, 115.99)	-2.83 (-3.13, -2.52)
Lower respiratory infections	Male	76536.60 (65950.49, 90742.86)	34346.41 (29779.62, 40082.51)	254.88 (219.58, 302.12)	118.62 (102.93, 138.38)	-2.71 (-3.05, -2.36)
Lower respiratory infections	Female	59460.71 (49210.54, 69519.36)	24014.53 (18045.01, 29425.92)	208.02 (172.16, 243.25)	86.42 (64.82, 105.93)	-2.98 (-3.26, -2.70)
Upper respiratory infections	Both	97387.58 (58515.82, 150587.11)	96853.23 (56083.43, 150901.64)	171.66 (102.84, 265.91)	164.55 (95.57, 255.95)	-0.54 (-0.68, -0.41)
Upper respiratory infections	Male	49843.14 (29867.19, 76378.90)	49593.59 (28722.59, 77011.55)	171.42 (102.40, 263.21)	164.80 (95.77, 255.57)	-0.55 (-0.67, -0.42)
Upper respiratory infections	Female	47544.44 (28566.91, 74134.77)	47259.64 (27333.38, 74247.06)	171.92 (102.98, 268.48)	164.28 (95.26, 257.65)	-0.54 (-0.68, -0.40)
Otitis media	Both	18479.69 (10633.51, 30733.95)	18911.14 (10809.20, 31533.09)	32.70 (18.82, 54.30)	32.00 (18.29, 53.46)	-0.11 (-0.15, -0.07)
Otitis media	Male	9994.93 (5803.34, 16589.40)	10138.75 (5815.27, 17186.78)	34.50 (20.00, 57.21)	33.57 (19.26, 57.01)	-0.12 (-0.17, -0.08)
Otitis media	Female	8484.75 (4865.02, 14006.72)	8772.39 (4946.51, 14582.56)	30.81 (17.68, 50.76)	30.36 (17.09, 50.57)	-0.10 (-0.13, -0.06)
Enteric infections	Both	40757.23 (30809.11, 53528.11)	11641.67 (9918.09, 13703.07)	69.65 (52.40, 91.92)	20.72 (17.67, 24.37)	-4.09 (-4.50, -3.67)
Enteric infections	Male	22433.45 (17072.47, 29233.17)	6222.49 (5067.68, 7490.66)	74.83 (56.70, 97.97)	21.68 (17.67, 26.07)	-4.25 (-4.70, -3.80)
Enteric infections	Female	18323.78 (13729.53, 24465.81)	5419.18 (4233.91, 6807.10)	64.21 (47.88, 86.18)	19.73 (15.42, 24.77)	-3.87 (-4.27, -3.48)
Neglected tropical diseases and malaria	Both	8055.54 (3909.54, 16556.86)	8492.23 (5763.62, 12688.48)	14.29 (6.89, 29.50)	14.37 (9.80, 21.40)	0.37 (0.21, 0.52)

**Disease**	**Sex**	**DALYs in 1990**	**DALYs in 2023**	**ASDR in 1990 (per 100,000)**	**ASDR in 2023 (per 100,000)**	**EAPC (95% CI)**
Neglected tropical diseases and malaria	Male	3935.60 (1925.29, 8340.98)	4388.60 (2364.83, 7467.11)	13.52 (6.58, 28.71)	14.60 (7.93, 24.70)	0.73 (0.54, 0.93)
Neglected tropical diseases and malaria	Female	4119.94 (1852.11, 8212.99)	4103.63 (2174.94, 7397.93)	15.10 (6.73, 30.15)	14.13 (7.57, 25.30)	0.01 (-0.12, 0.14)
Other infectious diseases	Both	114454.81 (103816.70, 126838.11)	54795.42 (47892.37, 62843.12)	196.55 (178.15, 218.15)	95.73 (83.83, 109.46)	-2.18 (-2.46, -1.90)
Other infectious diseases	Male	63097.89 (55997.17, 71108.24)	29881.51 (25137.63, 34688.28)	211.34 (187.54, 238.43)	102.29 (86.21, 118.51)	-2.16 (-2.45, -1.87)
Other infectious diseases	Female	51356.92 (45058.37, 59310.31)	24913.90 (20630.93, 29790.67)	181.04 (158.72, 209.32)	88.87 (73.69, 105.91)	-2.20 (-2.48, -1.92)

Table A.4: Age-specific burden of infectious diseases incidence among children in the United States

Disease	Sex	Number_1990_<5 years	Number_2023_<5 years	Number_1990_5-9 years	Number_2023_5-9 years	Number_1990_10-14 years	Number_2023_10-14 years	Rate_1990_<5 years	Rate_2023_<5 years	Rate_1990_5-9 years	Rate_2023_5-9 years	Rate_1990_10-14 years	Rate_2023_10-14 years
Acute hepatitis	Male	383968.84 (313760.96, 478477.56)	388156.69 (312199.00, 483569.81)	278730.07 (225132.38, 337077.95)	324152.08 (261857.87, 388997.06)	210482.19 (174994.84, 250949.69)	263422.56 (222792.31, 303990.46)	3798.17 (3103.68, 4733.04)	4099.47 (3297.25, 5107.17)	2932.14 (2368.31, 3545.93)	3136.24 (2533.53, 3763.63)	2331.68 (1938.56, 2779.98)	2445.26 (2068.10, 2821.83)
Acute hepatitis	Female	382001.87 (309081.01, 477675.92)	387631.59 (309288.06, 482372.29)	268588.91 (217003.84, 325163.90)	317032.34 (259716.47, 377476.04)	199075.58 (166177.20, 233322.56)	254203.26 (215337.78, 293008.34)	3962.33 (3205.96, 4954.71)	4278.69 (3413.93, 5324.44)	2966.70 (2396.92, 3591.60)	3211.54 (2630.93, 3823.83)	2318.73 (1935.55, 2717.62)	2479.21 (2100.16, 2857.67)
Acute hepatitis	Both	765970.71 (627711.50, 958498.14)	775788.28 (620582.09, 969606.95)	547318.98 (440778.30, 663405.85)	641184.42 (522841.38, 767614.39)	409557.78 (342890.41, 481167.42)	517625.82 (440743.94, 596515.09)	3878.30 (3178.26, 4853.12)	4187.10 (3349.42, 5233.18)	2949.00 (2374.95, 3574.48)	3173.02 (2587.38, 3798.69)	2325.37 (1946.85, 2731.95)	2461.81 (2096.16, 2837.01)
HIV/AIDS	Male	5612.67 (3683.09, 7892.16)	1162.70 (659.14, 1693.28)	0.00 (0.00, 0.00)	0.00 (0.00, 0.00)	0.00 (0.00, 0.00)	0.00 (0.00, 0.00)	55.52 (36.43, 78.07)	12.28 (6.96, 17.88)	0.00 (0.00, 0.00)	0.00 (0.00, 0.00)	0.00 (0.00, 0.00)	0.00 (0.00, 0.00)
HIV/AIDS	Female	5170.54 (3460.18, 7205.73)	1073.57 (605.41, 1544.80)	0.00 (0.00, 0.00)	0.00 (0.00, 0.00)	0.00 (0.00, 0.00)	0.00 (0.00, 0.00)	53.63 (35.89, 74.74)	11.85 (6.68, 17.05)	0.00 (0.00, 0.00)	0.00 (0.00, 0.00)	0.00 (0.00, 0.00)	0.00 (0.00, 0.00)
HIV/AIDS	Both	10783.21 (7143.05, 15088.57)	2236.28 (1278.82, 3235.28)	0.00 (0.00, 0.00)	0.00 (0.00, 0.00)	0.00 (0.00, 0.00)	0.00 (0.00, 0.00)	54.60 (36.17, 76.40)	12.07 (6.90, 17.46)	0.00 (0.00, 0.00)	0.00 (0.00, 0.00)	0.00 (0.00, 0.00)	0.00 (0.00, 0.00)
Sexually transmitted infections excluding HIV	Male	1372.96 (854.96, 2043.86)	1060.43 (675.19, 1562.14)	16.48 (2.36, 49.53)	17.38 (2.76, 51.87)	59342.65 (42432.45, 84236.51)	69083.72 (51279.04, 97328.08)	13.58 (8.46, 20.22)	11.20 (7.13, 16.50)	0.17 (0.02, 0.52)	0.17 (0.03, 0.50)	657.39 (470.06, 933.16)	641.28 (476.01, 903.46)
Sexually transmitted infections excluding HIV	Female	1318.85 (820.45, 1979.69)	982.96 (621.25, 1452.80)	15.60 (1.82, 46.51)	16.76 (2.05, 49.59)	75608.72 (56643.09, 96631.14)	89440.16 (69517.84, 112836.22)	13.68 (8.51, 20.53)	10.85 (6.86, 16.04)	0.17 (0.02, 0.51)	0.17 (0.02, 0.50)	880.65 (659.75, 1125.51)	872.30 (678.00, 1100.48)

Disease	Sex	Number_1990_<5 years	Number_2023_<5 years	Number_1990_5-9 years	Number_2023_5-9 years	Number_1990_10-14 years	Number_2023_10-14 years	Rate_1990_<5 years	Rate_2023_<5 years	Rate_1990_5-9 years	Rate_2023_5-9 years	Rate_1990_10-14 years	Rate_2023_10-14 years
Sexually transmitted infections excluding HIV	Both	2691.80 (1675.68, 4008.98)	2043.39 (1297.56, 3031.55)	32.08 (4.99, 95.72)	34.14 (5.65, 101.01)	134951.37 (102893.15, 177736.52)	158523.87 (123481.93, 203837.62)	13.63 (8.48, 20.30)	11.03 (7.00, 16.36)	0.17 (0.03, 0.52)	0.17 (0.03, 0.50)	766.22 (584.20, 1009.14)	753.93 (587.28, 969.45)
Tuberculosis	Male	170.74 (127.04, 224.67)	97.53 (74.46, 128.08)	81.54 (45.90, 131.87)	49.76 (29.01, 84.97)	76.75 (40.49, 138.42)	65.05 (33.41, 116.56)	1.69 (1.26, 2.22)	1.03 (0.79, 1.35)	0.86 (0.48, 1.39)	0.48 (0.28, 0.82)	0.85 (0.45, 1.53)	0.60 (0.31, 1.08)
Tuberculosis	Female	182.58 (130.42, 246.41)	65.63 (48.30, 88.90)	160.21 (97.46, 255.50)	51.15 (30.83, 80.08)	154.22 (87.99, 253.24)	63.38 (35.64, 106.55)	1.89 (1.35, 2.56)	0.72 (0.53, 0.98)	1.77 (1.08, 2.82)	0.52 (0.31, 0.81)	1.80 (1.02, 2.95)	0.62 (0.35, 1.04)
Tuberculosis	Both	353.33 (265.63, 473.46)	163.16 (123.14, 217.76)	241.75 (147.20, 377.80)	100.91 (60.27, 164.16)	230.97 (130.22, 396.54)	128.44 (68.80, 224.34)	1.79 (1.34, 2.40)	0.88 (0.66, 1.18)	1.30 (0.79, 2.04)	0.50 (0.30, 0.81)	1.31 (0.74, 2.25)	0.61 (0.33, 1.07)
Lower respiratory infections	Male	154327.82 (131880.56, 178721.39)	97634.42 (83532.73, 113365.64)	40158.03 (28440.89, 56980.19)	32122.61 (23728.26, 42651.13)	22506.52 (15033.26, 30888.32)	20629.65 (14854.45, 26402.75)	1526.59 (1304.54, 1767.89)	1031.16 (882.22, 1197.30)	422.45 (299.19, 599.41)	310.79 (229.58, 412.66)	249.32 (166.54, 342.18)	191.50 (137.89, 245.09)
Lower respiratory infections	Female	125406.76 (107426.46, 145797.85)	77788.30 (66476.04, 91170.91)	36258.87 (25263.06, 50760.60)	28371.31 (21084.24, 38031.41)	22121.19 (14934.90, 30203.87)	18908.37 (13801.63, 24296.29)	1300.79 (1114.29, 1512.29)	858.63 (733.76, 1006.35)	400.50 (279.04, 560.68)	287.40 (213.58, 385.26)	257.66 (173.95, 351.80)	184.41 (134.61, 236.96)
Lower respiratory infections	Both	279734.58 (239452.00, 324709.51)	175422.72 (150098.00, 204536.54)	76416.89 (54364.78, 107740.79)	60493.92 (45056.80, 80530.90)	44627.71 (29968.16, 61142.17)	39538.03 (28753.52, 50874.22)	1416.37 (1212.41, 1644.09)	946.80 (810.11, 1103.93)	411.74 (292.92, 580.52)	299.37 (222.97, 398.52)	253.39 (170.15, 347.15)	188.04 (136.75, 241.96)
Upper respiratory infections	Male	63142176.08 (53087966.20, 74105005.83)	58716808.32 (49524040.29, 69548753.82)	39473167.39 (28242921.57, 52262212.43)	42981086.97 (30529528.74, 57007338.69)	28588556.17 (19983541.47, 39097577.01)	34278923.58 (23919578.24, 46219711.07)	624593.90 (525139.01, 733036.73)	620131.01 (523042.62, 734531.39)	415242.86 (297104.90, 549778.80)	415850.96 (295379.55, 551557.87)	316698.99 (221374.15, 433116.07)	318199.01 (222036.90, 429041.08)

Disease	Sex	Number_1990_<5 years	Number_2023_<5 years	Number_1990_5-9 years	Number_2023_5-9 years	Number_1990_10-14 years	Number_2023_10-14 years	Rate_1990_<5 years	Rate_2023_<5 years	Rate_1990_5-9 years	Rate_2023_5-9 years	Rate_1990_10-14 years	Rate_2023_10-14 years
Upper respiratory infections	Female	59650178.71 (50330654.71, 70597102.43)	55999359.54 (46939237.42, 66023313.10)	38066816.79 (27292949.04, 50405172.12)	41307935.99 (29296086.44, 54900945.48)	29285926.62 (20833045.08, 39798386.93)	33603499.47 (23878838.37, 45413326.08)	618723.86 (522056.72, 732271.26)	618122.08 (518116.27, 728766.69)	420467.14 (301464.35, 556750.48)	418449.50 (296769.43, 556146.72)	341107.39 (242652.58, 463551.11)	327729.97 (232886.79, 442909.46)
Upper respiratory infections	Both	122792354.78 (103164061.47, 144727523.58)	114716167.86 (96338430.33, 136258537.55)	77539984.19 (55064092.73, 102667384.55)	84289022.96 (60117790.78, 111908284.18)	57874482.78 (41102273.15, 79041143.70)	67882423.06 (47898315.71, 92200350.97)	621728.50 (522345.52, 732791.76)	619148.71 (519959.97, 735417.68)	417791.30 (296689.50, 553179.63)	417120.40 (297504.42, 553799.61)	328597.26 (233368.73, 448776.42)	322846.77 (227802.96, 438502.11)
Otitis media	Male	2337826.64 (1827675.58, 2977814.87)	2320047.29 (1834333.06, 2897032.22)	736782.67 (518275.70, 995926.68)	846018.03 (605103.38, 1113079.77)	266072.52 (195378.96, 358180.19)	340768.49 (255966.20, 452124.40)	23125.47 (18079.12, 29456.14)	24502.92 (19373.10, 30596.68)	7750.68 (5452.07, 10476.77)	8185.40 (5854.50, 10769.28)	2947.50 (2164.37, 3967.86)	3163.23 (2376.04, 4196.91)
Otitis media	Female	1992537.70 (1533489.73, 2529764.61)	1988249.65 (1564630.20, 2475125.08)	695623.95 (487253.09, 945644.35)	806494.68 (594746.69, 1060846.64)	266509.22 (193944.80, 368781.43)	340396.68 (251674.68, 461201.74)	20667.68 (15906.18, 26240.08)	21946.34 (17270.42, 27320.48)	7683.52 (5381.96, 10445.12)	8169.79 (6024.79, 10746.38)	3104.16 (2258.97, 4295.38)	3319.84 (2454.55, 4498.03)
Otitis media	Both	4330364.34 (3366027.39, 5504958.34)	4308296.94 (3403193.38, 5383093.32)	1432406.62 (1010120.47, 1945262.02)	1652512.71 (1200390.12, 2168280.03)	532581.74 (392011.29, 722591.37)	681165.17 (507973.73, 896143.54)	21925.72 (17043.04, 27872.99)	23252.84 (18367.79, 29053.75)	7717.92 (5442.61, 10481.22)	8177.78 (5940.36, 10730.15)	3023.87 (2225.74, 4102.70)	3239.60 (2415.91, 4262.03)
Enteric infections	Male	3708947.72 (2691020.19, 4995258.79)	365199.50 (289825.00, 455244.45)	1028922.45 (513134.62, 1726092.33)	57877.99 (32376.76, 88766.55)	545850.14 (269099.31, 997908.13)	37368.81 (20314.87, 63225.16)	36688.41 (26619.21, 49412.43)	3857.01 (3060.95, 4808.01)	10823.88 (5397.98, 18157.84)	559.98 (313.25, 858.83)	6046.83 (2981.03, 11054.65)	346.88 (188.58, 586.90)
Enteric infections	Female	3343086.91 (2419925.29, 4505718.07)	351983.15 (275242.62, 434059.80)	927884.57 (470155.39, 1553241.25)	52216.11 (28691.53, 80299.45)	529407.32 (266099.25, 964870.10)	36188.77 (19870.82, 61218.51)	34676.30 (25100.77, 46735.74)	3885.20 (3038.13, 4791.16)	10248.95 (5193.10, 17156.33)	528.95 (290.65, 813.43)	6166.26 (3099.39, 11238.31)	352.94 (193.80, 597.06)
Enteric infections	Both	7052034.63 (5082564.21, 9618784.01)	717182.64 (562272.90, 893587.05)	1956807.02 (977099.20, 3223568.41)	110094.11 (61125.19, 168858.16)	1075257.46 (540872.09, 1955918.41)	73557.58 (40185.69, 123666.72)	35706.22 (25734.30, 48702.32)	3870.79 (3034.71, 4822.89)	10543.42 (5264.68, 17368.83)	544.82 (302.49, 835.63)	6105.05 (3070.94, 11105.23)	349.84 (191.12, 588.16)
Neglected tropical diseases and malaria	Male	2.73 (1.90, 3.93)	8.27 (6.04, 11.44)	7.76 (3.91, 13.33)	16.07 (11.05, 22.20)	13.05 (6.73, 25.92)	20.06 (13.70, 28.95)	0.03 (0.02, 0.04)	0.09 (0.06, 0.12)	0.08 (0.04, 0.14)	0.16 (0.11, 0.21)	0.14 (0.07, 0.29)	0.19 (0.13, 0.27)

Disease	Sex	Number_1990_<5 years	Number_2023_<5 years	Number_1990_5-9 years	Number_2023_5-9 years	Number_1990_10-14 years	Number_2023_10-14 years	Rate_1990_<5 years	Rate_2023_<5 years	Rate_1990_5-9 years	Rate_2023_5-9 years	Rate_1990_10-14 years	Rate_2023_10-14 years
Neglected tropical diseases and malaria	Female	2.26 (1.60, 3.06)	8.24 (6.00, 11.11)	4.99 (2.80, 8.01)	14.98 (10.53, 20.91)	7.18 (3.78, 13.53)	17.49 (12.08, 24.64)	0.02 (0.02, 0.03)	0.09 (0.07, 0.12)	0.06 (0.03, 0.09)	0.15 (0.11, 0.21)	0.08 (0.04, 0.16)	0.17 (0.12, 0.24)
Neglected tropical diseases and malaria	Both	4.99 (3.49, 6.82)	16.51 (12.04, 22.56)	12.74 (6.87, 20.74)	31.04 (21.56, 43.17)	20.23 (10.44, 39.94)	37.54 (26.11, 53.61)	0.03 (0.02, 0.03)	0.09 (0.06, 0.12)	0.07 (0.04, 0.11)	0.15 (0.11, 0.21)	0.11 (0.06, 0.23)	0.18 (0.12, 0.25)
Other infectious diseases	Male	1379782.12 (1206634.01, 1518023.43)	662263.43 (571442.40, 762548.37)	354145.92 (273759.54, 458995.91)	359871.04 (299274.59, 426489.04)	226755.67 (189570.33, 266094.01)	280801.35 (240128.98, 320112.45)	13648.62 (11935.86, 15016.08)	6994.42 (6035.23, 8053.57)	3725.48 (2879.85, 4828.46)	3481.83 (2895.54, 4126.37)	2511.96 (2100.03, 2947.74)	2606.58 (2229.03, 2971.49)
Other infectious diseases	Female	1331781.74 (1157164.18, 1482997.66)	667153.32 (576436.14, 763845.87)	350553.30 (270258.03, 462198.39)	357734.27 (295251.33, 418248.87)	216793.70 (181440.28, 255195.18)	273435.89 (236420.24, 311467.05)	13813.96 (12002.73, 15382.45)	7364.05 (6362.71, 8431.35)	3872.04 (2985.14, 5105.21)	3623.85 (2990.90, 4236.86)	2525.10 (2113.32, 2972.38)	2666.78 (2305.77, 3037.69)
Other infectious diseases	Both	2711563.86 (2370907.68, 2999863.81)	1329416.75 (1147682.00, 1521961.61)	704699.22 (547025.04, 919730.92)	717605.31 (593479.61, 842610.02)	443549.37 (370618.75, 521508.69)	554237.24 (478840.95, 630070.56)	13729.33 (12004.50, 15189.06)	7175.16 (6194.30, 8214.37)	3796.97 (2947.41, 4955.58)	3551.21 (2936.95, 4169.82)	2518.37 (2104.28, 2961.00)	2635.94 (2277.35, 2996.60)

Table A.5: Age-specific burden of infectious disease mortality among children in the United States

Disease	Sex	Number_1990_<5 years	Number_2023_<5 years	Number_1990_5-9 years	Number_2023_5-9 years	Number_1990_10-14 years	Number_2023_10-14 years	Rate_1990_<5 years	Rate_2023_<5 years	Rate_1990_5-9 years	Rate_2023_5-9 years	Rate_1990_10-14 years	Rate_2023_10-14 years
Acute hepatitis	Male	6.18 (4.32, 8.96)	2.06 (1.42, 3.21)	0.36 (0.25, 0.51)	0.14 (0.09, 0.20)	0.38 (0.26, 0.55)	0.16 (0.10, 0.23)	0.06 (0.04, 0.09)	0.02 (0.01, 0.03)	0.00 (0.00, 0.01)	0.00 (0.00, 0.00)	0.00 (0.00, 0.01)	0.00 (0.00, 0.00)
Acute hepatitis	Female	5.51 (3.68, 7.64)	1.37 (0.87, 1.96)	0.46 (0.32, 0.62)	0.14 (0.09, 0.19)	0.56 (0.40, 0.75)	0.20 (0.13, 0.27)	0.06 (0.04, 0.08)	0.02 (0.01, 0.02)	0.01 (0.00, 0.01)	0.00 (0.00, 0.00)	0.01 (0.00, 0.01)	0.00 (0.00, 0.00)
Acute hepatitis	Both	11.69 (8.94, 14.98)	3.43 (2.55, 4.65)	0.82 (0.64, 1.04)	0.28 (0.21, 0.36)	0.95 (0.74, 1.17)	0.35 (0.27, 0.46)	0.06 (0.05, 0.08)	0.02 (0.01, 0.03)	0.00 (0.00, 0.01)	0.00 (0.00, 0.00)	0.01 (0.00, 0.01)	0.00 (0.00, 0.00)
HIV/AIDS	Male	153.30 (140.31, 165.68)	12.88 (11.29, 14.53)	35.09 (29.75, 40.15)	3.86 (3.11, 4.61)	24.62 (21.31, 28.12)	3.88 (2.89, 4.70)	1.52 (1.39, 1.64)	0.14 (0.12, 0.15)	0.37 (0.31, 0.42)	0.04 (0.03, 0.04)	0.27 (0.24, 0.31)	0.04 (0.03, 0.04)
HIV/AIDS	Female	119.14 (108.06, 131.41)	12.68 (10.48, 14.37)	25.29 (21.35, 29.17)	4.04 (3.29, 4.79)	13.56 (11.63, 15.36)	3.78 (3.07, 4.42)	1.24 (1.12, 1.36)	0.14 (0.12, 0.16)	0.28 (0.24, 0.32)	0.04 (0.03, 0.05)	0.16 (0.14, 0.18)	0.04 (0.03, 0.04)
HIV/AIDS	Both	272.44 (253.54, 291.09)	25.56 (22.91, 28.24)	60.38 (54.11, 66.14)	7.90 (6.87, 9.14)	38.18 (34.49, 41.94)	7.66 (6.53, 8.71)	1.38 (1.28, 1.47)	0.14 (0.12, 0.15)	0.33 (0.29, 0.36)	0.04 (0.03, 0.05)	0.22 (0.20, 0.24)	0.04 (0.03, 0.04)
Sexually transmitted infections excluding HIV	Male	6.40 (4.81, 8.24)	1.53 (1.10, 1.98)	0.00 (0.00, 0.00)	0.00 (0.00, 0.00)	0.37 (0.25, 0.52)	0.10 (0.07, 0.16)	0.06 (0.05, 0.08)	0.02 (0.01, 0.02)	0.00 (0.00, 0.00)	0.00 (0.00, 0.00)	0.00 (0.00, 0.01)	0.00 (0.00, 0.00)
Sexually transmitted infections excluding HIV	Female	5.82 (4.16, 7.90)	1.29 (0.83, 1.79)	0.12 (0.09, 0.16)	0.03 (0.02, 0.04)	0.30 (0.24, 0.39)	0.19 (0.15, 0.25)	0.06 (0.04, 0.08)	0.01 (0.01, 0.02)	0.00 (0.00, 0.00)	0.00 (0.00, 0.00)	0.00 (0.00, 0.00)	0.00 (0.00, 0.00)
Sexually transmitted infections	Both	12.22 (9.85, 14.34)	2.82 (2.25, 3.45)	0.12 (0.09, 0.16)	0.03 (0.02, 0.04)	0.67 (0.54, 0.84)	0.30 (0.24, 0.37)	0.06 (0.05, 0.07)	0.02 (0.01, 0.02)	0.00 (0.00, 0.00)	0.00 (0.00, 0.00)	0.00 (0.00, 0.00)	0.00 (0.00, 0.00)

Disease	Sex	Number_1990_<5 years	Number_2023_<5 years	Number_1990_5-9 years	Number_2023_5-9 years	Number_1990_10-14 years	Number_2023_10-14 years	Rate_1990_<5 years	Rate_2023_<5 years	Rate_1990_5-9 years	Rate_2023_5-9 years	Rate_1990_10-14 years	Rate_2023_10-14 years
excluding HIV													
Tuberculosis	Male	7.57 (6.30, 9.15)	1.20 (0.97, 1.44)	1.44 (1.20, 1.73)	0.25 (0.20, 0.29)	1.28 (1.06, 1.55)	0.29 (0.24, 0.34)	0.07 (0.06, 0.09)	0.01 (0.01, 0.02)	0.02 (0.01, 0.02)	0.00 (0.00, 0.00)	0.01 (0.01, 0.02)	0.00 (0.00, 0.00)
Tuberculosis	Female	8.25 (6.90, 9.78)	1.25 (0.95, 1.56)	1.83 (1.49, 2.21)	0.35 (0.28, 0.44)	1.62 (1.32, 1.94)	0.48 (0.39, 0.60)	0.09 (0.07, 0.10)	0.01 (0.01, 0.02)	0.02 (0.02, 0.02)	0.00 (0.00, 0.00)	0.02 (0.02, 0.02)	0.00 (0.00, 0.01)
Tuberculosis	Both	15.82 (13.86, 18.04)	2.45 (2.08, 2.80)	3.27 (2.84, 3.73)	0.60 (0.50, 0.69)	2.90 (2.51, 3.28)	0.76 (0.64, 0.89)	0.08 (0.07, 0.09)	0.01 (0.01, 0.02)	0.02 (0.02, 0.02)	0.00 (0.00, 0.00)	0.02 (0.01, 0.02)	0.00 (0.00, 0.00)
Lower respiratory infections	Male	757.70 (654.28, 901.35)	311.58 (272.46, 362.74)	58.67 (49.43, 67.93)	42.11 (35.23, 49.66)	48.27 (40.44, 56.03)	37.81 (31.15, 45.23)	7.50 (6.47, 8.92)	3.29 (2.88, 3.83)	0.62 (0.52, 0.71)	0.41 (0.34, 0.48)	0.53 (0.45, 0.62)	0.35 (0.29, 0.42)
Lower respiratory infections	Female	578.42 (478.24, 675.32)	208.28 (152.68, 256.98)	49.81 (42.17, 58.69)	35.02 (28.55, 41.66)	45.05 (36.74, 53.63)	31.22 (25.34, 38.33)	6.00 (4.96, 7.00)	2.30 (1.69, 2.84)	0.55 (0.47, 0.65)	0.35 (0.29, 0.42)	0.52 (0.43, 0.62)	0.30 (0.25, 0.37)
Lower respiratory infections	Both	1336.12 (1196.55, 1495.17)	519.86 (448.66, 584.14)	108.48 (96.03, 120.46)	77.12 (67.94, 87.89)	93.32 (82.12, 104.84)	69.03 (59.53, 79.73)	6.77 (6.06, 7.57)	2.81 (2.42, 3.15)	0.58 (0.52, 0.65)	0.38 (0.34, 0.43)	0.53 (0.47, 0.60)	0.33 (0.28, 0.38)
Upper respiratory infections	Male	28.78 (19.74, 39.37)	9.33 (6.55, 13.09)	3.11 (2.11, 4.27)	1.14 (0.79, 1.66)	1.66 (1.19, 2.22)	0.74 (0.53, 1.01)	0.28 (0.20, 0.39)	0.10 (0.07, 0.14)	0.03 (0.02, 0.04)	0.01 (0.01, 0.02)	0.02 (0.01, 0.02)	0.01 (0.00, 0.01)
Upper respiratory infections	Female	21.54 (15.06, 30.08)	4.77 (3.18, 6.97)	2.98 (2.13, 4.03)	0.80 (0.53, 1.17)	1.43 (1.03, 1.89)	0.48 (0.35, 0.69)	0.22 (0.16, 0.31)	0.05 (0.04, 0.08)	0.03 (0.02, 0.04)	0.01 (0.01, 0.01)	0.02 (0.01, 0.02)	0.00 (0.00, 0.01)
Upper respiratory infections	Both	50.32 (39.27, 63.12)	14.10 (10.66, 18.50)	6.09 (4.75, 7.60)	1.94 (1.51, 2.56)	3.09 (2.48, 3.78)	1.22 (0.97, 1.56)	0.25 (0.20, 0.32)	0.08 (0.06, 0.10)	0.03 (0.03, 0.04)	0.01 (0.01, 0.01)	0.02 (0.01, 0.02)	0.01 (0.00, 0.01)

Disease	Sex	Number_1990_<5 years	Number_2023_<5 years	Number_1990_5-9 years	Number_2023_5-9 years	Number_1990_10-14 years	Number_2023_10-14 years	Rate_1990_<5 years	Rate_2023_<5 years	Rate_1990_5-9 years	Rate_2023_5-9 years	Rate_1990_10-14 years	Rate_2023_10-14 years
Otitis media	Male	6.05 (1.69, 12.59)	2.02 (0.71, 6.14)	0.47 (0.12, 0.99)	0.20 (0.07, 0.58)	0.34 (0.09, 0.75)	0.16 (0.05, 0.44)	0.06 (0.02, 0.12)	0.02 (0.01, 0.06)	0.00 (0.00, 0.01)	0.00 (0.00, 0.01)	0.00 (0.00, 0.01)	0.00 (0.00, 0.00)
Otitis media	Female	3.11 (0.67, 9.48)	0.82 (0.17, 2.79)	0.19 (0.04, 0.58)	0.06 (0.01, 0.21)	0.37 (0.07, 1.12)	0.12 (0.02, 0.41)	0.03 (0.01, 0.10)	0.01 (0.00, 0.03)	0.00 (0.00, 0.01)	0.00 (0.00, 0.00)	0.00 (0.00, 0.01)	0.00 (0.00, 0.00)
Otitis media	Both	9.16 (3.11, 16.92)	2.85 (1.14, 7.07)	0.66 (0.23, 1.24)	0.27 (0.11, 0.67)	0.71 (0.24, 1.43)	0.29 (0.08, 0.61)	0.05 (0.02, 0.09)	0.02 (0.01, 0.04)	0.00 (0.00, 0.01)	0.00 (0.00, 0.00)	0.00 (0.00, 0.01)	0.00 (0.00, 0.00)
Enteric infections	Male	135.17 (113.84, 163.10)	53.87 (43.33, 66.09)	1.83 (1.34, 2.63)	3.09 (2.31, 4.09)	1.27 (0.74, 2.38)	2.81 (1.97, 3.88)	1.34 (1.13, 1.61)	0.57 (0.46, 0.70)	0.02 (0.01, 0.03)	0.03 (0.02, 0.04)	0.01 (0.01, 0.03)	0.03 (0.02, 0.04)
Enteric infections	Female	99.78 (82.65, 123.06)	45.80 (34.34, 57.99)	1.50 (1.05, 2.29)	2.86 (2.08, 3.81)	1.00 (0.60, 1.86)	2.43 (1.79, 3.47)	1.03 (0.86, 1.28)	0.51 (0.38, 0.64)	0.02 (0.01, 0.03)	0.03 (0.02, 0.04)	0.01 (0.01, 0.02)	0.02 (0.02, 0.03)
Enteric infections	Both	234.95 (206.52, 268.05)	99.66 (84.16, 116.81)	3.34 (2.52, 4.49)	5.95 (5.00, 7.23)	2.27 (1.44, 3.70)	5.25 (4.13, 6.77)	1.19 (1.05, 1.36)	0.54 (0.45, 0.63)	0.02 (0.01, 0.02)	0.03 (0.02, 0.04)	0.01 (0.01, 0.02)	0.02 (0.02, 0.03)
Neglected tropical diseases and malaria	Male	8.56 (6.20, 12.11)	10.70 (7.22, 14.98)	1.60 (1.15, 2.27)	2.21 (1.53, 3.01)	1.44 (1.04, 1.95)	2.32 (1.69, 3.05)	0.08 (0.06, 0.12)	0.11 (0.08, 0.16)	0.02 (0.01, 0.02)	0.02 (0.01, 0.03)	0.02 (0.01, 0.02)	0.02 (0.02, 0.03)
Neglected tropical diseases and malaria	Female	7.02 (5.03, 9.51)	9.29 (6.70, 12.96)	1.36 (0.93, 1.90)	2.02 (1.38, 3.01)	1.04 (0.74, 1.40)	1.84 (1.39, 2.48)	0.07 (0.05, 0.10)	0.10 (0.07, 0.14)	0.02 (0.01, 0.02)	0.02 (0.01, 0.03)	0.01 (0.01, 0.02)	0.02 (0.01, 0.02)
Neglected tropical diseases and malaria	Both	15.58 (12.35, 20.01)	19.99 (15.21, 26.66)	2.96 (2.27, 3.81)	4.22 (3.14, 5.66)	2.47 (2.00, 3.11)	4.17 (3.34, 5.25)	0.08 (0.06, 0.10)	0.11 (0.08, 0.14)	0.02 (0.01, 0.02)	0.02 (0.02, 0.03)	0.01 (0.01, 0.02)	0.02 (0.02, 0.02)
Other infectious diseases	Male	561.45 (497.33, 627.84)	222.95 (190.47, 252.92)	62.68 (56.23, 71.12)	31.13 (26.66, 36.33)	41.71 (37.16, 48.74)	26.22 (22.72, 30.47)	5.55 (4.92, 6.21)	2.35 (2.01, 2.67)	0.66 (0.59, 0.75)	0.30 (0.26, 0.35)	0.46 (0.41, 0.54)	0.24 (0.21, 0.28)

Disease	Sex	Number_1990_<5 years	Number_2023_<5 years	Number_1990_5-9 years	Number_2023_5-9 years	Number_1990_10-14 years	Number_2023_10-14 years	Rate_1990_<5 years	Rate_2023_<5 years	Rate_1990_5-9 years	Rate_2023_5-9 years	Rate_1990_10-14 years	Rate_2023_10-14 years
Other infectious diseases	Female	439.69 (389.57, 500.25)	174.85 (145.78, 202.47)	49.53 (44.85, 55.52)	25.25 (22.38, 29.20)	42.49 (38.38, 47.08)	23.49 (20.73, 26.54)	4.56 (4.04, 5.19)	1.93 (1.61, 2.23)	0.55 (0.50, 0.61)	0.26 (0.23, 0.30)	0.49 (0.45, 0.55)	0.23 (0.20, 0.26)
Other infectious diseases	Both	1001.14 (913.25, 1086.37)	397.79 (354.64, 438.85)	112.21 (102.38, 122.33)	56.39 (51.32, 62.49)	84.20 (77.12, 91.80)	49.71 (45.26, 55.47)	5.07 (4.62, 5.50)	2.15 (1.91, 2.37)	0.60 (0.55, 0.66)	0.28 (0.25, 0.31)	0.48 (0.44, 0.52)	0.24 (0.22, 0.26)

Table A.6: Age-specific burden of DALYs among children in the United States

Disease	Sex	Number_1990_<5 years	Number_2023_<5 years	Number_1990_5-9 years	Number_2023_5-9 years	Number_1990_10-14 years	Number_2023_10-14 years	Rate_1990_<5 years	Rate_2023_<5 years	Rate_1990_5-9 years	Rate_2023_5-9 years	Rate_1990_10-14 years	Rate_2023_10-14 years
Acute hepatitis	Male	637.54 (455.75, 892.31)	268.46 (184.63, 385.37)	399.80 (243.09, 618.56)	456.01 (262.74, 753.64)	510.45 (335.34, 770.90)	650.15 (393.76, 1046.85)	6.31 (4.51, 8.83)	2.84 (1.95, 4.07)	4.21 (2.56, 6.51)	4.41 (2.54, 7.29)	5.65 (3.71, 8.54)	6.04 (3.66, 9.72)
Acute hepatitis	Female	577.49 (414.11, 783.67)	205.86 (146.00, 285.81)	403.41 (240.65, 657.82)	450.53 (259.78, 714.61)	510.65 (316.11, 813.05)	642.71 (397.92, 1038.84)	5.99 (4.30, 8.13)	2.27 (1.61, 3.15)	4.46 (2.66, 7.27)	4.56 (2.63, 7.24)	5.95 (3.68, 9.47)	6.27 (3.88, 10.13)
Acute hepatitis	Both	1215.03 (956.44, 1565.71)	474.33 (354.68, 618.35)	803.21 (486.53, 1258.70)	906.55 (564.67, 1447.43)	1021.10 (664.83, 1573.65)	1292.86 (807.31, 2052.39)	6.15 (4.84, 7.93)	2.56 (1.91, 3.34)	4.33 (2.62, 6.78)	4.49 (2.79, 7.16)	5.80 (3.77, 8.93)	6.15 (3.84, 9.76)
HIV/AIDS	Male	13688.10 (12537.13, 14788.66)	1198.83 (1048.24, 1340.53)	2922.00 (2477.23, 3339.08)	368.91 (304.08, 439.77)	1906.32 (1649.84, 2177.16)	338.40 (259.33, 406.61)	135.40 (124.02, 146.29)	12.66 (11.07, 14.16)	30.74 (26.06, 35.13)	3.57 (2.94, 4.25)	21.12 (18.28, 24.12)	3.14 (2.41, 3.77)
HIV/AIDS	Female	10658.09 (9664.77, 11761.42)	1178.30 (980.23, 1328.87)	2110.47 (1792.87, 2432.37)	382.05 (321.38, 452.13)	1052.21 (902.65, 1191.73)	329.90 (266.35, 385.22)	110.55 (100.25, 122.00)	13.01 (10.82, 14.67)	23.31 (19.80, 26.87)	3.87 (3.26, 4.58)	12.26 (10.51, 13.88)	3.22 (2.60, 3.76)
HIV/AIDS	Both	24346.18 (22694.92, 26018.30)	2377.14 (2138.04, 2616.50)	5032.47 (4519.35, 5508.58)	750.96 (652.32, 882.21)	2958.53 (2672.31, 3249.27)	668.30 (568.22, 762.62)	123.27 (114.91, 131.74)	12.83 (11.54, 14.12)	27.12 (24.35, 29.68)	3.72 (3.23, 4.37)	16.80 (15.17, 18.45)	3.18 (2.70, 3.63)
Sexually transmitted infections excluding HIV	Male	574.95 (432.14, 740.46)	137.51 (99.27, 178.33)	0.01 (0.00, 0.01)	0.00 (0.00, 0.01)	73.29 (48.06, 116.63)	60.06 (31.35, 114.96)	5.69 (4.27, 7.32)	1.45 (1.05, 1.88)	0.00 (0.00, 0.00)	0.00 (0.00, 0.00)	0.81 (0.53, 1.29)	0.56 (0.29, 1.07)
Sexually transmitted infections excluding HIV	Female	523.37 (373.57, 709.67)	116.25 (74.83, 160.51)	10.11 (7.44, 13.33)	2.24 (1.48, 3.04)	114.49 (72.23, 186.92)	122.38 (73.30, 194.20)	5.43 (3.87, 7.36)	1.28 (0.83, 1.77)	0.11 (0.08, 0.15)	0.02 (0.01, 0.03)	1.33 (0.84, 2.18)	1.19 (0.71, 1.89)

Disease	Sex	Number_1990_<5 years	Number_2023_<5 years	Number_1990_5-9 years	Number_2023_5-9 years	Number_1990_10-14 years	Number_2023_10-14 years	Rate_1990_<5 years	Rate_2023_<5 years	Rate_1990_5-9 years	Rate_2023_5-9 years	Rate_1990_10-14 years	Rate_2023_10-14 years
Sexually transmitted infections excluding HIV	Both	1098.32 (884.96, 1288.59)	253.76 (202.70, 310.38)	10.12 (7.45, 13.34)	2.25 (1.48, 3.05)	187.78 (125.96, 304.09)	182.44 (108.10, 318.35)	5.56 (4.48, 6.52)	1.37 (1.09, 1.68)	0.05 (0.04, 0.07)	0.01 (0.01, 0.02)	1.07 (0.72, 1.73)	0.87 (0.51, 1.51)
Tuberculosis	Male	718.58 (605.00, 869.52)	160.19 (133.12, 193.89)	137.37 (114.49, 164.29)	38.00 (28.89, 51.96)	107.64 (88.76, 130.07)	35.77 (26.85, 50.54)	7.11 (5.98, 8.60)	1.69 (1.41, 2.05)	1.45 (1.20, 1.73)	0.37 (0.28, 0.50)	1.19 (0.98, 1.44)	0.33 (0.25, 0.47)
Tuberculosis	Female	783.48 (654.57, 918.68)	154.47 (124.24, 184.32)	191.16 (157.92, 230.22)	56.37 (41.58, 76.84)	140.57 (113.24, 168.33)	51.83 (39.53, 66.57)	8.13 (6.79, 9.53)	1.71 (1.37, 2.03)	2.11 (1.74, 2.54)	0.57 (0.42, 0.78)	1.64 (1.32, 1.96)	0.51 (0.39, 0.65)
Tuberculosis	Both	1502.07 (1328.21, 1699.32)	314.67 (262.43, 367.29)	328.54 (280.98, 388.19)	94.36 (71.50, 127.65)	248.22 (209.17, 289.40)	87.60 (67.93, 116.16)	7.61 (6.73, 8.60)	1.70 (1.42, 1.98)	1.77 (1.51, 2.09)	0.47 (0.35, 0.63)	1.41 (1.19, 1.64)	0.42 (0.32, 0.55)
Lower respiratory infections	Male	67854.97 (58627.57, 80688.93)	27867.20 (24383.66, 32409.81)	4913.17 (4145.01, 5686.81)	3529.10 (2966.14, 4150.11)	3768.47 (3177.91, 4367.12)	2950.11 (2429.82, 3522.59)	671.21 (579.94, 798.16)	294.32 (257.53, 342.29)	51.68 (43.60, 59.82)	34.14 (28.70, 40.15)	41.75 (35.20, 48.38)	27.38 (22.56, 32.70)
Lower respiratory infections	Female	51761.48 (42802.91, 60425.33)	18634.79 (13671.12, 22949.64)	4175.70 (3533.13, 4902.63)	2936.93 (2394.20, 3486.32)	3523.53 (2874.50, 4191.40)	2442.81 (1979.69, 2989.96)	536.90 (443.97, 626.76)	205.69 (150.90, 253.32)	46.12 (39.03, 54.15)	29.75 (24.25, 35.32)	41.04 (33.48, 48.82)	23.82 (19.31, 29.16)
Lower respiratory infections	Both	119616.44 (107106.59, 133811.80)	46501.99 (40156.74, 52243.70)	9088.87 (8028.17, 10094.96)	6466.04 (5705.15, 7368.97)	7292.00 (6413.45, 8184.80)	5392.92 (4657.60, 6210.10)	605.65 (542.31, 677.52)	250.98 (216.73, 281.97)	48.97 (43.26, 54.39)	32.00 (28.23, 36.47)	41.40 (36.41, 46.47)	25.65 (22.15, 29.54)
Upper respiratory infections	Male	25126.96 (16007.10, 36893.29)	21829.21 (13469.24, 32918.61)	14502.90 (8407.15, 22817.51)	15600.43 (8847.32, 24793.78)	10213.29 (5452.93, 16668.10)	12163.95 (6406.03, 19299.16)	248.55 (158.34, 364.94)	230.55 (142.25, 347.67)	152.57 (88.44, 240.03)	150.94 (85.60, 239.89)	113.14 (60.41, 184.65)	112.91 (59.46, 179.15)
Upper respirator	Female	23231.73 (14841.07, 34923.76)	20457.78 (12439.35, 30906.85)	13960.54 (8074.64, 22443.81)	14969.25 (8559.55, 24125.92)	10352.17 (5651.20, 16767.21)	11832.61 (6334.48, 19214.28)	240.97 (153.94, 362.25)	225.81 (137.31, 341.15)	154.20 (89.19, 247.90)	151.64 (86.71, 244.40)	120.58 (65.82, 195.30)	115.40 (61.78, 187.39)

Disease	Sex	Number_1990_<5 years	Number_2023_<5 years	Number_1990_5-9 years	Number_2023_5-9 years	Number_1990_10-14 years	Number_2023_10-14 years	Rate_1990_<5 years	Rate_2023_<5 years	Rate_1990_5-9 years	Rate_2023_5-9 years	Rate_1990_10-14 years	Rate_2023_10-14 years
y infections													
Upper respiratory infections	Both	48358.68 (30783.55, 71817.05)	42286.99 (25917.91, 63510.08)	28463.44 (16590.08, 45346.66)	30569.68 (17440.48, 48919.71)	20565.46 (11142.19, 33423.41)	23996.55 (12725.03, 38471.85)	244.85 (155.86, 363.63)	228.23 (139.88, 342.78)	153.36 (89.39, 244.33)	151.28 (86.31, 242.09)	116.77 (63.26, 189.77)	114.13 (60.52, 182.97)
Otitis media	Male	4575.32 (2750.73, 7703.89)	4071.44 (2371.52, 7206.01)	3235.50 (1814.77, 5395.51)	3524.30 (1983.21, 5797.42)	2184.11 (1237.84, 3489.99)	2543.01 (1460.55, 4183.36)	45.26 (27.21, 76.21)	43.00 (25.05, 76.11)	34.04 (19.09, 56.76)	34.10 (19.19, 56.09)	24.20 (13.71, 38.66)	23.61 (13.56, 38.83)
Otitis media	Female	3688.00 (2083.90, 6372.97)	3390.99 (1839.73, 5925.72)	2813.21 (1620.13, 4537.94)	3084.05 (1768.00, 4972.28)	1983.54 (1160.99, 3095.82)	2297.34 (1338.79, 3684.56)	38.25 (21.62, 66.10)	37.43 (20.31, 65.41)	31.07 (17.90, 50.12)	31.24 (17.91, 50.37)	23.10 (13.52, 36.06)	22.41 (13.06, 35.93)
Otitis media	Both	8263.33 (4751.78, 14164.41)	7462.44 (4274.27, 12993.34)	6048.71 (3439.72, 9947.89)	6608.35 (3726.94, 10713.34)	4167.65 (2442.01, 6621.64)	4840.35 (2807.99, 7826.41)	41.84 (24.06, 71.72)	40.28 (23.07, 70.13)	32.59 (18.53, 53.60)	32.70 (18.44, 53.02)	23.66 (13.87, 37.60)	23.02 (13.35, 37.22)
Enteric infections	Male	19258.36 (15590.62, 23530.81)	5571.57 (4595.44, 6630.26)	2099.92 (985.30, 3615.63)	365.49 (271.25, 476.19)	1075.17 (496.55, 2086.73)	285.43 (200.99, 384.21)	190.50 (154.22, 232.76)	58.84 (48.53, 70.02)	22.09 (10.37, 38.04)	3.54 (2.62, 4.61)	11.91 (5.50, 23.12)	2.65 (1.87, 3.57)
Enteric infections	Female	15433.34 (12342.66, 19140.34)	4830.46 (3800.33, 6047.02)	1877.70 (912.16, 3334.78)	334.91 (244.38, 421.24)	1012.74 (474.71, 1990.69)	253.81 (189.21, 338.83)	160.08 (128.02, 198.53)	53.32 (41.95, 66.75)	20.74 (10.08, 36.83)	3.39 (2.48, 4.27)	11.80 (5.53, 23.19)	2.48 (1.85, 3.30)
Enteric infections	Both	34691.70 (27979.07, 42638.86)	10402.03 (8956.72, 12125.22)	3977.62 (1861.32, 6797.47)	700.40 (545.82, 872.54)	2087.91 (968.72, 4091.78)	539.23 (415.56, 705.31)	175.65 (141.67, 215.89)	56.14 (48.34, 65.44)	21.43 (10.03, 36.63)	3.47 (2.70, 4.32)	11.85 (5.50, 23.23)	2.56 (1.98, 3.35)
Neglected tropical diseases and malaria	Male	2112.98 (1131.11, 4331.10)	2029.05 (1262.10, 3056.76)	975.12 (456.03, 2005.29)	1202.43 (554.24, 2218.14)	847.50 (338.14, 2004.60)	1157.12 (548.49, 2192.21)	20.90 (11.19, 42.84)	21.43 (13.33, 32.28)	10.26 (4.80, 21.09)	11.63 (5.36, 21.46)	9.39 (3.75, 22.21)	10.74 (5.09, 20.35)
Neglected tropical diseases	Female	1528.11 (819.93, 2939.73)	1478.46 (970.76, 2246.97)	1210.20 (545.18, 2423.69)	1229.17 (647.84, 2292.91)	1381.63 (487.00, 2849.57)	1396.00 (556.33, 2858.05)	15.85 (8.50, 30.49)	16.32 (10.72, 24.80)	13.37 (6.02, 26.77)	12.45 (6.56, 23.23)	16.09 (5.67, 33.19)	13.61 (5.43, 27.87)

Disease	Sex	Number_1990_<5 years	Number_2023_<5 years	Number_1990_5-9 years	Number_2023_5-9 years	Number_1990_10-14 years	Number_2023_10-14 years	Rate_1990_<5 years	Rate_2023_<5 years	Rate_1990_5-9 years	Rate_2023_5-9 years	Rate_1990_10-14 years	Rate_2023_10-14 years
and malaria													
Neglected tropical diseases and malaria	Both	3641.09 (2015.01, 6790.12)	3507.52 (2629.60, 4886.09)	2185.32 (1013.97, 4611.83)	2431.60 (1549.34, 3708.64)	2229.13 (880.55, 5154.92)	2553.11 (1584.68, 4093.75)	18.44 (10.20, 34.38)	18.93 (14.19, 26.37)	11.77 (5.46, 24.85)	12.03 (7.67, 18.35)	12.66 (5.00, 29.27)	12.14 (7.54, 19.47)
Other infectious diseases	Male	51640.68 (45868.23, 57443.91)	21748.13 (18723.40, 24623.03)	6646.32 (5930.53, 7737.87)	4243.91 (3390.71, 5200.28)	4810.88 (4198.41, 5926.46)	3889.47 (3023.52, 4864.98)	510.82 (453.72, 568.23)	229.69 (197.75, 260.05)	69.92 (62.39, 81.40)	41.06 (32.81, 50.31)	53.29 (46.51, 65.65)	36.10 (28.07, 45.16)
Other infectious diseases	Female	40507.94 (35869.95, 46128.18)	17280.79 (14550.63, 19704.93)	5679.93 (4853.55, 6733.37)	3792.09 (3063.41, 4943.58)	5169.05 (4334.88, 6448.76)	3841.03 (3016.90, 5142.16)	420.17 (372.06, 478.47)	190.75 (160.61, 217.50)	62.74 (53.61, 74.37)	38.41 (31.03, 50.08)	60.21 (50.49, 75.11)	37.46 (29.42, 50.15)
Other infectious diseases	Both	92148.62 (84362.92, 100163.92)	39028.91 (34960.88, 43020.33)	12326.26 (10893.85, 14380.89)	8036.00 (6696.10, 9948.19)	9979.93 (8559.93, 12293.29)	7730.50 (6235.38, 9874.60)	466.57 (427.15, 507.16)	210.65 (188.69, 232.19)	66.41 (58.70, 77.49)	39.77 (33.14, 49.23)	56.66 (48.60, 69.80)	36.77 (29.66, 46.96)

Table A.7: Segmented log-linear regression analysis of age-standardized disease rates

Disease	Outcome	No. Segments	Period	APC (%)	Direction
Enteric infections	Incidence	3	1990-1995	-24.14	Decreasing
			1995-2002	+5.77	Increasing
			2002-2015	-21.49	Decreasing
			2015-2023	+20.71	Increasing
Enteric infections	Mortality	3	1990-1999	+2.34	Increasing
			1999-2004	+10.73	Increasing
			2004-2011	+0.31	Increasing
			2011-2023	-4.61	Decreasing
Enteric infections	DALYs	3	1990-1993	-9.59	Decreasing
			1993-2002	+1.14	Increasing
			2002-2020	-4.50	Decreasing
			2020-2023	+3.90	Increasing
Lower respiratory infections	Incidence	3	1990-1995	+1.82	Increasing
			1995-2002	-2.72	Decreasing
			2002-2011	+0.23	Increasing
			2011-2023	-3.39	Decreasing
Lower respiratory infections	Mortality	2	1990-1998	-0.93	Decreasing
			1998-1999	-15.94	Decreasing
			1999-2023	-1.94	Decreasing
Lower respiratory infections	DALYs	3	1990-1998	-2.56	Decreasing
			1998-1999	-10.49	Decreasing
			1999-2021	-1.91	Decreasing
			2021-2023	+5.21	Increasing

Upper respiratory infections	Incidence	3	1990-2001	+0.23	Increasing
			2001-2014	-1.39	Decreasing
			2014-2021	+0.28	Increasing
			2021-2023	+4.89	Increasing
Upper respiratory infections	Mortality	3	1990-2000	-9.50	Decreasing
			2000-2015	-0.76	Decreasing
			2015-2019	-3.63	Decreasing
			2019-2023	+2.48	Increasing
Upper respiratory infections	DALYs	3	1990-2002	-0.08	Decreasing
			2002-2014	-1.33	Decreasing
			2014-2021	+0.22	Increasing
			2021-2023	+4.63	Increasing
Neglected tropical diseases and malaria	Incidence	0	1990-2023	+3.25	Increasing
Neglected tropical diseases and malaria	Mortality	3	1990-1999	-2.26	Decreasing
			1999-2003	+13.22	Increasing
			2003-2009	-1.29	Decreasing
			2009-2023	+1.69	Increasing
Neglected tropical diseases and malaria	DALYs	3	1990-1996	-1.24	Decreasing
			1996-2004	+1.13	Increasing
			2004-2009	-0.98	Decreasing
			2009-2023	+0.04	Increasing

Figure A.1: Segmented Regression – Incidence Rate U.S. Children 0-14 Years

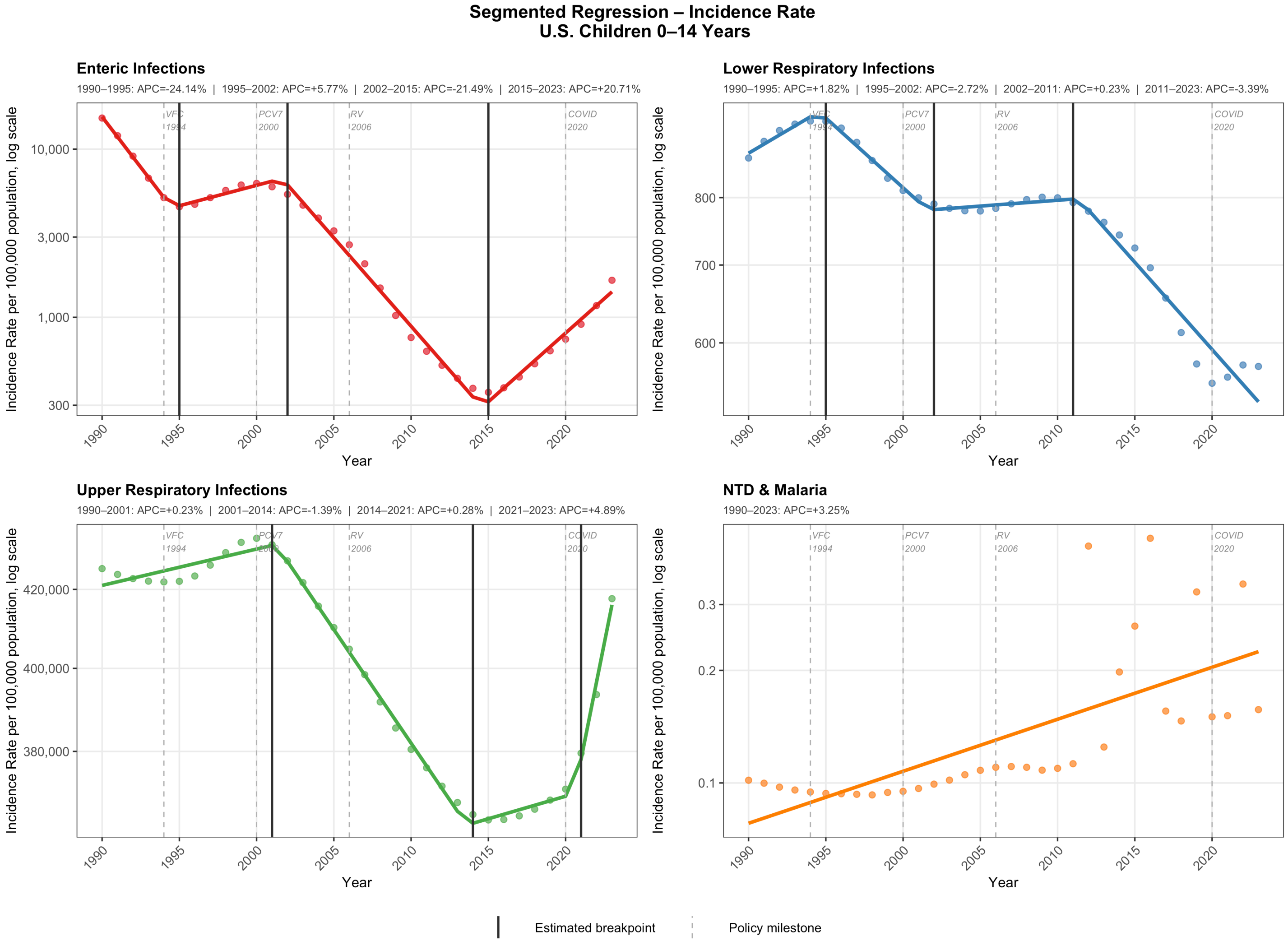

Legend: Age-standardized incidence rates and fitted segmented regression trends for selected infectious disease categories. Vertical dashed lines mark contextual milestones and were not modeled as causal exposures.

Figure A.2: Segmented Regression – Mortality Rate U.S. Children 0-14 Years

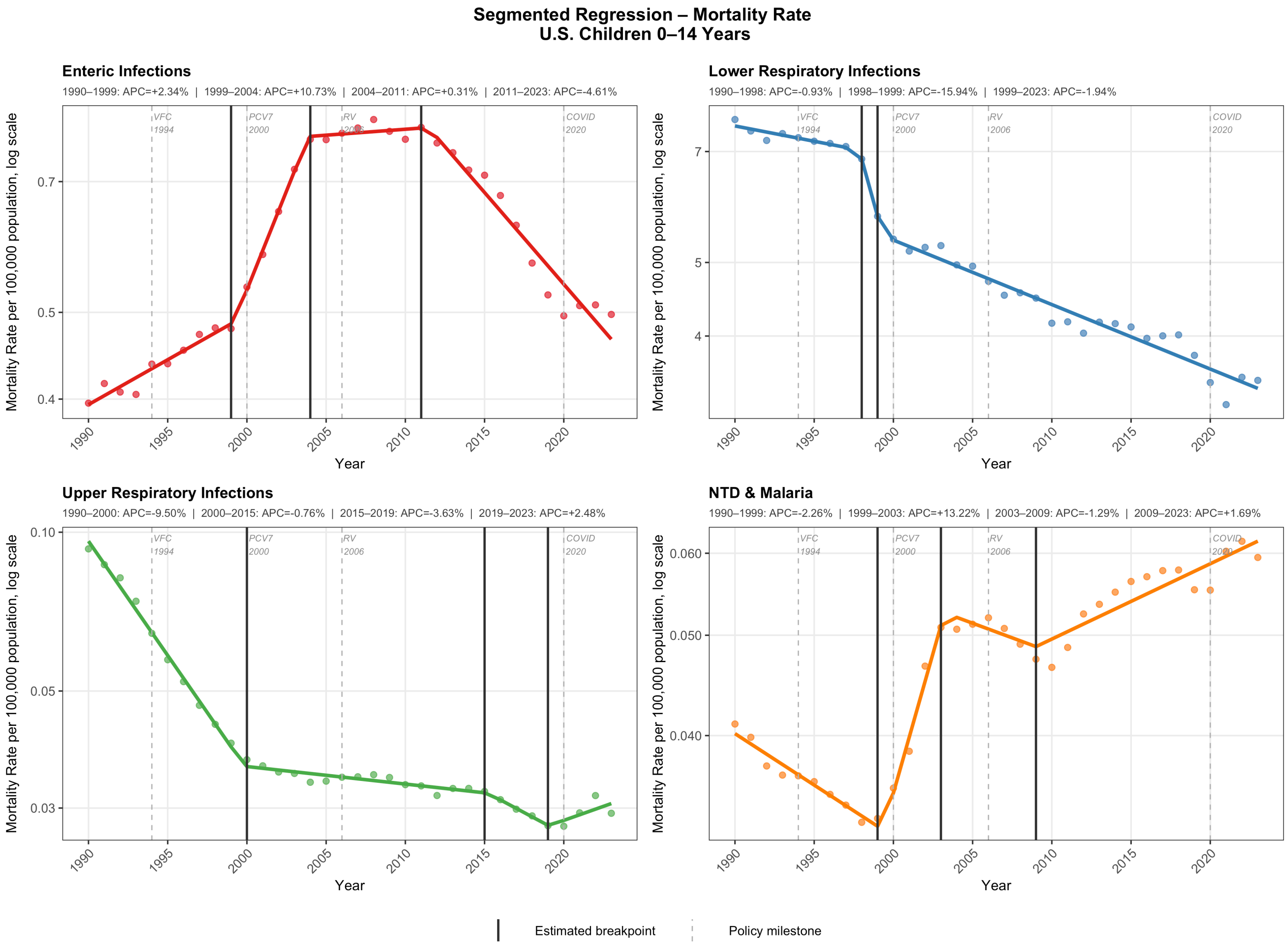

Legend: Age-standardized mortality rates and fitted segmented regression trends for selected infectious disease categories. Vertical dashed lines mark contextual milestones and were not modeled as causal exposures.

Figure A.3: Segmented Regression – DALYs Rate U.S. Children 0-14 Years

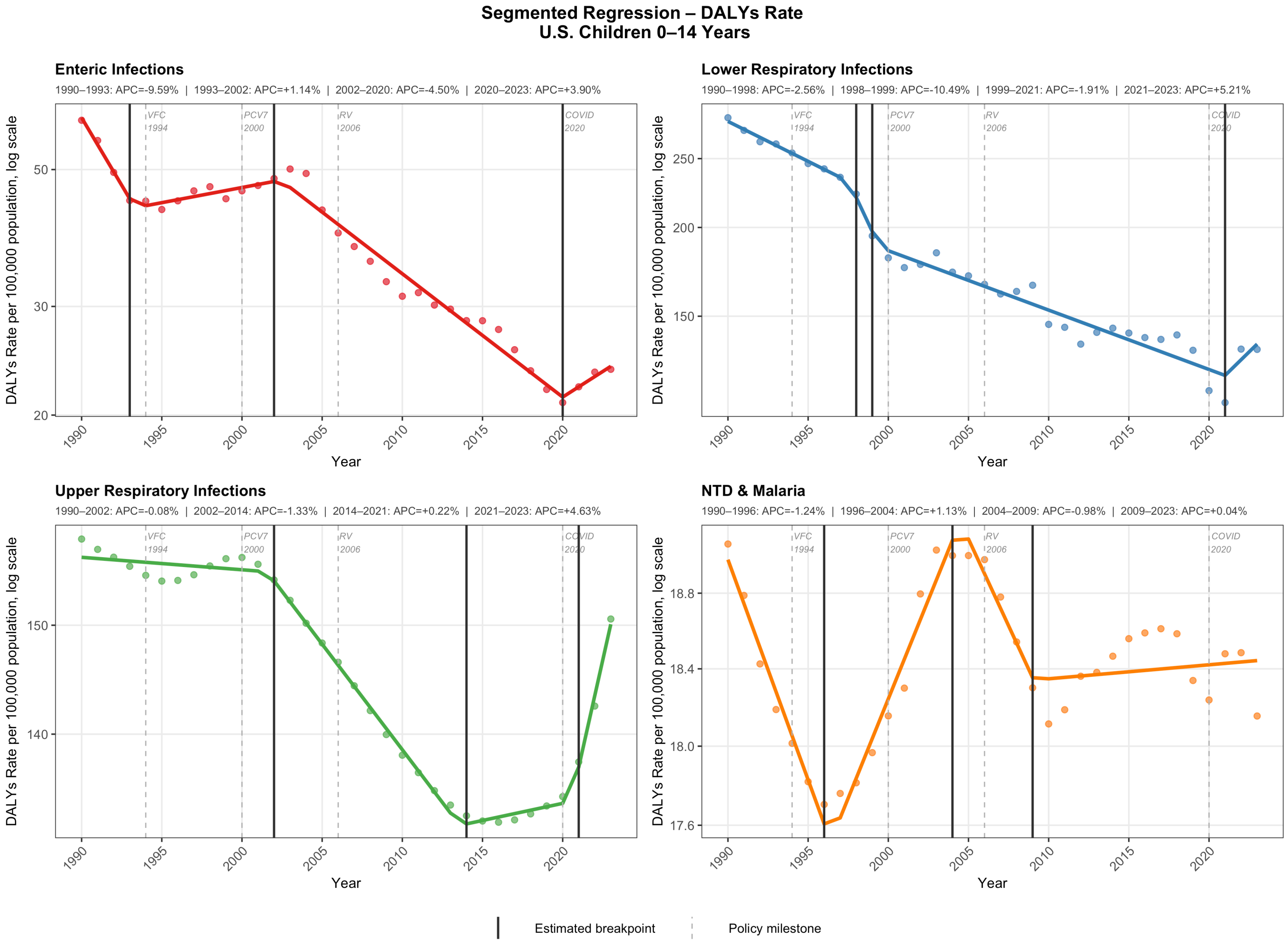

Legend: Age-standardized disability-adjusted life-year rates and fitted segmented regression trends for selected infectious disease categories. Vertical dashed lines mark contextual milestones and were not modeled as causal exposures.
